# Uveal Melanoma Cell Line Proliferation Is Inhibited by Ricolinostat, a Histone Deacetylase Inhibitor

**DOI:** 10.3390/cancers14030782

**Published:** 2022-02-03

**Authors:** Husvinee Sundaramurthi, Sandra García-Mulero, Valentina Tonelotto, Kayleigh Slater, Simone Marcone, Josep M. Piulats, Ronald William Watson, Desmond J. Tobin, Lasse D. Jensen, Breandán N. Kennedy

**Affiliations:** 1 UCD Conway Institute, University College Dublin, D04 V1W8 Dublin, Ireland; husvinee.sundaramurthi@ucd.ie (H.S.); valentina.tonelotto@ucd.ie (V.T.); kayleigh.slater@ucdconnect.ie (K.S.); william.watson@ucd.ie (R.W.W.); desmond.tobin@ucd.ie (D.J.T.); 2 UCD School of Biomolecular and Biomedical Science, University College Dublin, D04 V1W8 Dublin, Ireland; 3 Systems Biology Ireland, University College Dublin, D04 V1W8 Dublin, Ireland; 4 UCD School of Medicine, University College Dublin, D04 V1W8 Dublin, Ireland; 5 Cancer Immunotherapy (CIT) Group—iPROCURE, Program in Molecular Mechanisms and Experimental Therapy in Oncology (Oncobell), IDIBELL, L’Hospitalet de Llobregat, 08908 Barcelona, Spain; s.garciam@idibell.cat (S.G.-M.); jmpiulats@iconcologia.net (J.M.P.); 6 Unit of Biomarkers and Susceptibility, Oncology Data Analytics Program (ODAP), Catalan Institute of Oncology, L’Hospitalet de Llobregat, 08908 Barcelona, Spain; 7 Xenopat S.L., Business Bioincubator, Bellvitge Health Science Campus, L’Hospitalet de Llobregat, 08907 Barcelona, Spain; 8 Trinity Translational Medicine Institute, Department of Surgery, Trinity College Dublin St. James’s Hospital, D08 W9RT Dublin, Ireland; marcones@tcd.ie; 9 Medical Oncology Department, Catalan Institute of Cancer (ICO) and CIBERONC, L’Hospitalet de Llobregat, 08908 Barcelona, Spain; 10 The Charles Institute for Dermatology, School of Medicine, University College Dublin, D04 V1W8 Dublin, Ireland; 11 BioReperia AB, Wahlbecksgatan, 582 16 Linköping, Sweden; lasse.jensen@bioreperia.com

**Keywords:** metastatic uveal melanoma, HDAC inhibitor, ACY-1215, MITF, p-ERK, ML329, zebrafish xenografts

## Abstract

**Simple Summary:**

Uveal melanoma (UM) is the most common adult eye cancer. UM originates in the iris, ciliary body or choroid (collectively known as the uvea), in the middle layer of the eye. This first or primary UM is treated by targeting cancer cells using ocular radiation implants or by surgical removal of the eye. However, when UM spreads to the liver and other parts of the body, patients have a poor survival prognosis. Unfortunately, there are no effective treatment options for UM that has spread. Our aim is to help identify effective treatments for UM. In our study, we identified that the drug ACY-1215 prevents the growth of cells derived from UM in the eye and a UM that spread to the liver. Our pre-clinical study uncovered a potential treatment approach for advanced UM.

**Abstract:**

Metastatic uveal melanoma (MUM) is characterized by poor patient survival. Unfortunately, current treatment options demonstrate limited benefits. In this study, we evaluate the efficacy of ACY-1215, a histone deacetylase inhibitor (HDACi), to attenuate growth of primary ocular UM cell lines and, in particular, a liver MUM cell line in vitro and in vivo, and elucidate the underlying molecular mechanisms. A significant (*p* = 0.0001) dose-dependent reduction in surviving clones of the primary ocular UM cells, Mel270, was observed upon treatment with increasing doses of ACY-1215. Treatment of OMM2.5 MUM cells with ACY-1215 resulted in a significant (*p* = 0.0001), dose-dependent reduction in cell survival and proliferation in vitro, and in vivo attenuation of primary OMM2.5 xenografts in zebrafish larvae. Furthermore, flow cytometry revealed that ACY-1215 significantly arrested the OMM2.5 cell cycle in S phase (*p* = 0.0001) following 24 h of treatment, and significant apoptosis was triggered in a time- and dose-dependent manner (*p* < 0.0001). Additionally, ACY-1215 treatment resulted in a significant reduction in OMM2.5 p-ERK expression levels. Through proteome profiling, the attenuation of the microphthalmia-associated transcription factor (MITF) signaling pathway was linked to the observed anti-cancer effects of ACY-1215. In agreement, pharmacological inhibition of MITF signaling with ML329 significantly reduced OMM2.5 cell survival and viability in vitro (*p* = 0.0001) and reduced OMM2.5 cells in vivo (*p* = 0.0006). Our findings provide evidence that ACY-1215 and ML329 are efficacious against growth and survival of OMM2.5 MUM cells.

## 1. Introduction

Uveal melanoma (UM) is the most common adult intraocular cancer, afflicting approximately 4.3 per million people worldwide [1]. Although a rare cancer, incidence rates increase geographically in a south–north gradient, with countries such as Ireland, Norway and Denmark reported to have the highest incidences in Europe [2,3,4]. UM originates with different frequencies in the uveal tract of the eye: in the choroid (~90%), iris (~4%) and ciliary body (~6%) [5]. The most effective treatments for primary UM include surgical resection of the tumor, radiotherapy (plaque brachytherapy or proton beam therapy) and enucleation of the affected eye [4,6]. Unfortunately, approximately 50% of patients diagnosed with primary UM progress to develop metastatic UM (MUM), primarily in the liver (~89%), which is associated with poor survival prognosis (median overall survival (OS) ranging from 4 to 15 months) [2,7,8]. There is no standard of care treatment for MUM patients, and current therapeutic options have limited benefit. MUM patients receive site -directed therapies (including surgical resection of tumor), the chemotherapeutic drug Dacarbazine (commonly used to treat cutaneous melanoma) or immunotherapy drugs, such as Ipilimumab and Pembrolizumab [8,9]. Unfortunately, treatment with Dacarbazine, either as a monotherapy or combinatorial therapy, does not improve overall survival or progression-free survival [8,10,11,12]. In addition, immune checkpoint inhibitors, MEK inhibitors and liver-directed therapies are in clinical and pre-clinical trials for UM at present [13,14,15]. Recently, a Phase III clinical study with Tebentafusp (a form of immunotherapy that recruits and redirects T cells to tumor cells) reported favorable evidence in MUM patients with the one-year OS rate reported at 73% (N = 252) in the Tebentafusp treatment group compared to 59% (N = 126) in the control group, with an estimated median OS of 21.7 months and 16.0 months, respectively [16]. Nevertheless, there is still an imperative to identify highly efficacious, sustained treatments for MUM patients, as Tebentafusp has only been trialed in a subset of MUM patient cohort who are HLA-A*02:01-positive.

Histone deacetylase inhibitors (HDACi) have garnered widespread interest in the past two decades as anti-cancer agents [17,18,19,20,21]. Four pan-HDAC inhibitors—Vorinostat (SAHA) for relapsed and refractory cutaneous T-cell lymphoma (CTCL), Belinostat for peripheral T-cell lymphoma (PTCL), Romidepsin for CTCL/PTCL and Panobinostat for multiple myeloma – are approved for market use as treatment options by the FDA and/or EMA [18]. Chidamide is approved by the Chinese FDA for treatment of PTCL, with more research underway in other cancers [22]. Pre-clinical studies identified pan-HDACi to show efficacy as anti-cancer agents in UM and/or MUM cell lines, in vitro and/or in vivo [23]. Encouragingly, the first Phase II clinical trial with 29 MUM patients reported that a combination treatment of Entinostat (pan-HDACi) and Pembrolizumab (PD-1 inhibitor) resulted in a median OS of 13.4 months, with one-year OS reported as 59%, and median progression-free survival (PFS) of 2.1 months and a 17% one-year PFS [24,25]. More recently, a novel compound, VS13, which displays increased selectivity against HDAC6, reduced UM cell viability [26].

In relation to selective HDAC inhibition, histone deacetylase 6 inhibitors (HDAC6i) have shown promise as anti-cancer agents in pre-clinical studies and are currently under clinical trial investigations as a monotherapy or combinatorial therapy for lymphoproliferative disease, hematologic malignancies and solid tumors [27,28,29,30]. HDAC6 is a Class IIb enzyme and, unlike other HDAC isozymes, mainly resides in the cytoplasm and acts primarily on cytosolic proteins [31]. This provides a potential selective advantage over pan-HDAC inhibitors due to their pleiotropic effects. Pre-clinical studies report multiple selective HDAC6i compounds as anti-cancer agents with anti-cell proliferation, anti-cell viability and tumor attenuation in glioblastoma, ovarian cancer and bladder cancer [17,30,32,33,34]. A handful of HDAC6i clinical trials are registered and currently proceeding. A Phase Ib/II trial of ACY-1215 (Ricolinostat) in a small cohort of lymphoma patients revealed it was well tolerated, and the disease stabilized in 50% (8 out of 16 patients evaluated) of patients [27]. ACY-1215 in combination with paclitaxel was well tolerated and exhibited activity in patients with ovarian cancer in a small-scale Phase Ib trial, which was prematurely terminated [28]. In a Phase I/II trial in patients with relapsed or refractory multiple myeloma, ACY-1215, given in combination with Bortezomib and Dexamethasone, was well tolerated and active as an anti-myeloma agent [29]. There are also ongoing clinical trials with HDAC6 inhibitors (e.g., ACY-1215, Citarinostat (ACY-241) or KA2507) as a single agent or combination therapy for non-small cell lung cancer, metastatic breast cancer and solid tumors [30].

Here, we investigated the efficacy of ACY-1215 in inhibiting growth of a primary ocular UM cell line and then focused on ACY-1215 effects on the viability and growth of the OMM2.5 MUM cell line and the underlying molecular mechanisms. ACY-1215 significantly attenuated growth of the MUM cell line, OMM2.5, and this effect correlated with reduced levels of microphthalmia-associated transcription factor (MITF).

## 2. Results

### 2.1. ACY-1215 Significantly Attenuates Long-Term Proliferation of Human Uveal Melanoma Cell Lines

**Figure 1:**
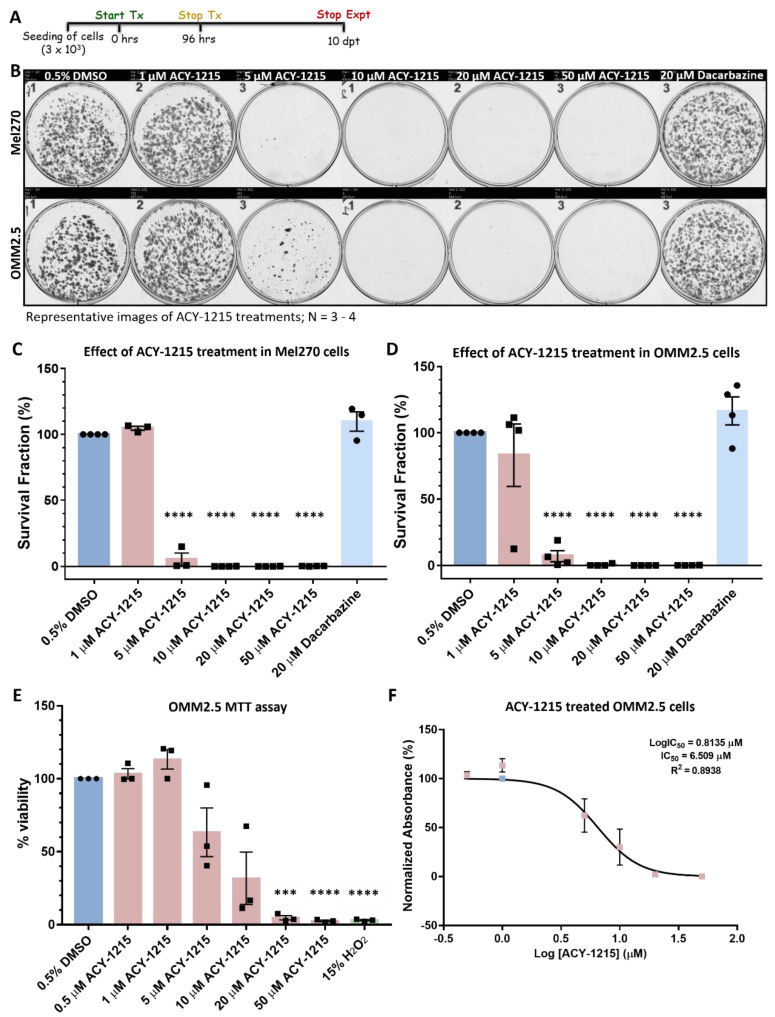
ACY-1215 is efficacious as an anti-cancer drug in primary UM and metastatic UM cell lines. (**A**): Schematic diagram on the treatment regime followed. Days post treatment (dpt). (**B**): Representative image of clonogenic assay plates for Mel270 (top panel) and OMM2.5 cells (bottom panel) treated with 0.5% DMSO; 1, 5, 10, 20 or 50 μM ACY-1215 or 20 μM Dacarbazine for 96 h. (**C**,**D**): A dose-dependent, significant decrease in the surviving number of OMM2.5 colonies was observed, indicative of reduction in cell viability upon ACY-1215 treatment in comparison to 0.5% DMSO treatment. (**E**): A dose-dependent, significant decrease in OMM2.5 cell viability was observed following 96 h of ACY-1215 treatment in comparison to 0.5% DMSO treatment. (**F**): IC_50_ value of ACY-1215 in OMM2.5 cell viability assays. One-way ANOVA with Dunnett’s Test for Multiple Comparisons statistical analysis was performed; error bars represent mean ± SEM, *** *p* value of 0.001, **** *p* value of 0.0001 (*N* = 3–4).

Three commercially available HDAC6i (Tubastatin A, ACY-1215 and Tubacin) were selected to determine their efficacy in reducing long-term proliferation of human UM cell lines derived from primary ocular UM (Mel285 and Mel270) and a metastatic liver (OMM2.5) UM [35,36,37]. Cells were treated for 96 h at selected concentrations; the treatment was stopped, cells were cultured for another 10 days in fresh complete media, and the colonies formed visualized with crystal violet staining and were counted [38]. Initial screens with 10–50 µM showed a dose-dependent reduction in UM cell proliferation of both primary UM cell lines and the metastatic UM cell line with all three HDAC6i tested (Figure S1). ACY-1215 was selected as the highest-ranked drug for subsequent studies based on its observed effects in all three UM cell lines tested and its existing approved use in clinical trials [27,28,29]. ACY-1215 was tested at 1, 5, 10, 20 and 50 µM concentrations in primary UM Mel270 and metastatic OMM2.5 cells, as these cell lines were established from the same patient (Figure 1). Mel270 cells showed significant reductions in viable clones, averaging 94.7%, 99.98%, 99.98% and 99.8% (*p* = 0.0001) decreases at 5, 10, 20 and 50 µM concentration of ACY-1215, respectively, compared to vehicle control (0.5% DMSO) (Figure 1B,C). Similarly, in OMM2.5 cells, ACY-1215 significantly reduced surviving colonies in a dose-dependent manner, averaging 92.9%, 99.5%, 99.98% and 99.8% (*p* = 0.0001) decreases when treated at 5, 10, 20 and 50 µM, respectively, compared to vehicle control (Figure 1B,D). Patients diagnosed with MUM were previously prescribed the chemotherapeutic Dacarbazine, hence this was used as a clinical control treatment on both Mel270 and OMM2.5 cells. However, there was no significant difference observed at the tested concentration of 20 µM in either Mel270 (9.8% increase in colony formation, *p* = 0.095) or OMM2.5 (16.5% increase in colony formation, *p* = 0.704) cells (Figure 1B–D). Additionally, the cell viability of ACY-1215-treated OMM2.5 cells was assessed and the IC_50_ value determined using an MTT assay (Figure 1E,F). OMM2.5 cells were treated with 0.5% DMSO (vehicle control) or increasing concentrations of ACY-1215 (0.5, 1, 5, 10, 20 and 50 µM) or 15% hydrogen peroxide (H_2_O_2_) for 96 h. We observed a significant, dose-dependent reduction in OMM2.5 cell viability when treated at 20 µM (95.34%, *p* = 0.0001) and 50 µM (97.48%, *p* = 0.0001) ACY-1215, compared to vehicle control (Figure 1E). Similarly, a significant (96.96%, *p* = 0.0001) reduction in OMM2.5 cell viability was observed when treated with 15% H_2_O_2_. There was no significant difference in cell viability observed when treated with 0.5, 1, 5 and 10 µM ACY-1215. From the MTT assay, the IC_50_ for ACY-1215 in OMM2.5 cells was calculated to be 6.51 µM (Figure 1F). As our primary goal was to identify potential novel treatment strategies for MUM, follow-on studies focused on ACY-1215 efficacy and the mechanism of action in OMM2.5 cells.

### 2.2. Zebrafish OMM2.5 Xenografts Proved That ACY-1215 Is Efficacious In Vivo

**Figure 2:**
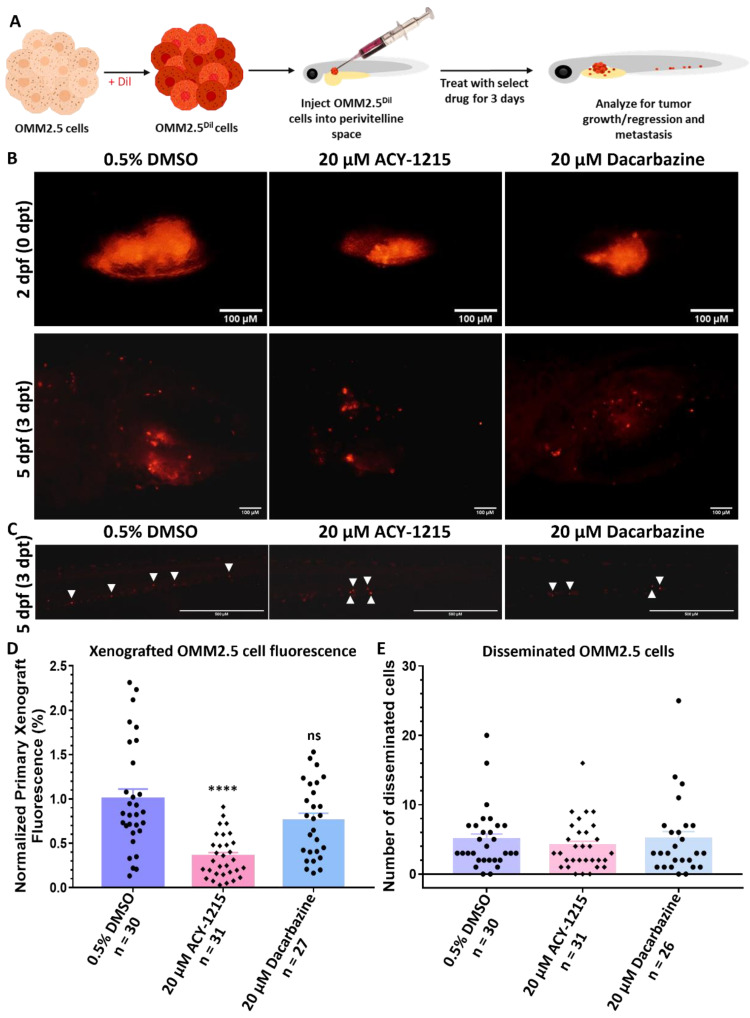
ACY-1215 demonstrates anti-cancer effects in vivo in zebrafish OMM2.5 xenografts. (**A**): Schematic depicting the workflow for assessing ACY-1215 effects in vivo. (**B**): Top panel shows representative images of Dil-labeled OMM2.5 cells transplanted into the perivitelline space of 2-day-old zebrafish larvae. Bottom panels present representative images of the distribution of OMM2.5 Dil-labeled cells in xenografts 3 days post treatment (dpt), with 0.5% DMSO (*n* = 30), 20 μM ACY-1215 (*n* = 31) or 20 μM Dacarbazine (*n* = 27). Days post fertilization (dpf). (**C**): At 3 dpt, OMM2.5 Dil-labeled cells have disseminated (white arrowhead) to the caudal vein plexus of the zebrafish larvae. (**D**): ACY-1215 treatment for 3 days resulted in a significant (****, *p* = 0.0001) reduction in normalized primary xenograft fluorescence on average in comparison to larvae treated with 0.5% DMSO or 20 μM Dacarbazine. (**E**): There was no observed difference in the average number of disseminated cells between vehicle control-treated or drug-treated groups after 3 days. Statistical analysis was performed using one-way ANOVA with Dunnett’s Test for Multiple Comparisons, and error bars present mean ± SEM.

Our in vitro study provided preliminary evidence that ACY-1215 has anti-UM properties. Therefore, the efficacy of ACY-1215 in vivo was evaluated using zebrafish OMM2.5 xenografts, a pre-clinical model of MUM. A toxicity screen determined the maximum tolerated dose of ACY-1215 and Dacarbazine in zebrafish larvae, with both drugs well tolerated at all tested concentrations (Figure S2). OMM2.5 cells labeled with Dil, a lipophilic membrane dye, were transplanted into the perivitelline space of 2-day-old larvae, and xenografts were treated with 0.5% DMSO, 20 µM ACY-1215 or 20 µM Dacarbazine for 3 days (5 days old) (Figure 2A). These concentrations were selected based on the in vitro studies conducted. Primary xenograft fluorescence from OMM2.5 transplants regressed by approximately 65% (*p* < 0.0001) with 20 µM ACY-1215 treatment compared to vehicle controls (Figure 2B,D). There was no notable difference in primary xenograft fluorescence when treated with 20 µM Dacarbazine in comparison to vehicle control. Additionally, the ability of transplanted OMM2.5 cells to disseminate was assessed by the number of cells present at the caudal vein plexus, 3 days post treatment. Dissemination of OMM2.5 Dil-labeled cells was not affected by either 20 µM ACY-1215 or 20 µM Dacarbazine (Figure 2C,E). On average, four disseminated OMM2.5 Dil-labeled cells were detected at the caudal vein plexus of ACY-1215-treated larvae, and five disseminated cells were counted in larvae treated with either 20 µM Dacarbazine or 0.5% DMSO. In summary, ACY-1215 at the tested concentration was effective in reducing OMM2.5 cell fluorescence intensity, but not the dissemination of OMM2.5 xenografts, in vivo.

### 2.3. Analysis of ACY-1215 Targets in UM Patient Samples and UM Cells

**Figure 3:**
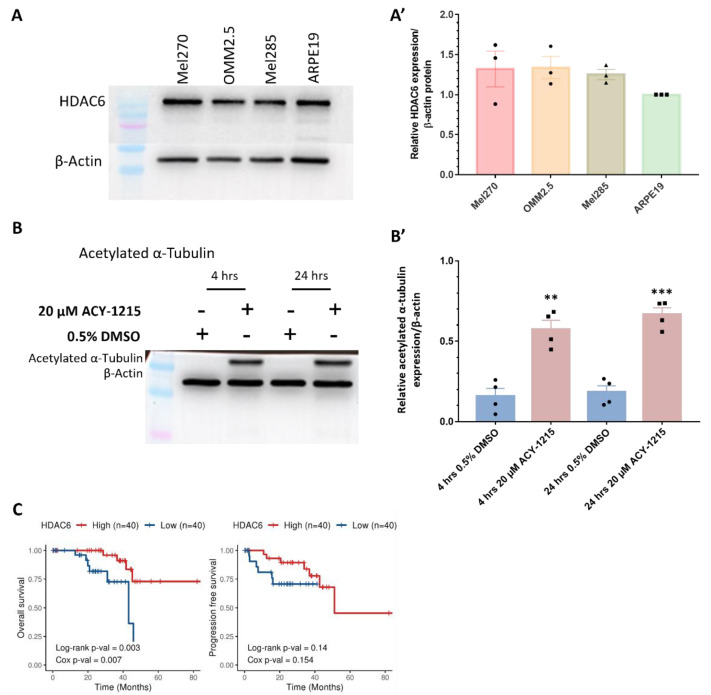
Expression and activity of HDAC6 in UM/MUM cells. (**A**,**A’**): HDAC6 is expressed in Mel270, OMM2.5, Mel285 and ARPE19 cells (N = 3). (**B**,**B’**): 20 μM ACY-1215 treatment significantly increased acetylated α-tubulin expression levels at 4 h post treatment (hpt) (**, *p* = 0.0013) and 24 hpt (***, *p* = 0.0002) compared to 0.5% DMSO treatment. Student’s Unpaired T test statistical analysis was performed, and data are presented as mean ± SEM. Representative blots for each protein probed and densitometry analysis presented, plus raw blots are provided in Supplementary Figures S3 and S7. (**C**): Kaplan–Meier survival curves assessing correlation between expression of HDAC6 and overall survival (OS) or progression-free survival (PFS) in UM patients. Median values were used as cut-off for high (red) and low (blue) expression levels, with Log-rank *p*-values (categorical variable) and Cox *p*-values (continuous variable) calculated (*n* = 80).

HDAC6 is a selective target of ACY-1215 at lower concentrations, hence HDAC6 expression in the different UM/MUM cell lines was confirmed by immunoblotting (Figure 3). No significant difference in HDAC6 expression was detected when the untreated primary ocular tumor-derived cell lines (Mel270 and Mel285) or untreated MUM (OMM2.5) cell line were compared to untreated ARPE19 cells, a human retinal pigment epithelium cell line (Figure 3A,A’ and Figure S3). To determine whether ACY-1215 was indeed blocking HDAC6 activity, the expression of its downstream substrate, acetylated α-tubulin, was analyzed [30]. We observed a significant increase in acetylated α-tubulin levels after 4 (3.56-fold increase, *p* = 0.001) and 24 (3.67-fold increase, *p* = 0.0002) hours post treatment (hpt) with 20 µM ACY-1215 compared to 0.5% DMSO-treated OMM2.5 cells, confirming the inhibitory effects of ACY-1215 (Figure 3B,B’ and Figure S7A). As a dose-dependent anti-cancer effect of ACY-1215 was observed in the clonogenic assays and zebrafish xenografts, correlations between expression level of *HDAC6* transcript expression and UM patient overall survival/progression-free survival were analyzed. Extracting the gene expression data of 80 primary UM samples from The Cancer Genome Atlas (TCGA), Cox proportional hazards models and Kaplan–Meier survival curves were generated. Kaplan–Meier survival curves were generated with a cut-off of 50% to demarcate a high or low *HDAC6* expression, and the Log-rank test was used to compare survival probability between the groups. Interestingly, high *HDAC6* transcript expression was significantly associated with better overall survival, but not with progression-free survival (Cox OS, *p* = 0.007 and Cox PFS, *p* = 0.154) (Figure 3C).

A known caveat of ACY-1215 is the non-selective inhibition of other HDAC isozymes at higher concentrations. The reported IC_50_ of ACY-1215 in an enzymatic-based assay is 4.7 nM, at which ACY-1215 acts as a highly potent and selective HDAC6 inhibitor [39]. Hence, we postulated that the observed effects of ACY-1215 in OMM2.5 cells are partly attributed to parallel inhibition of other HDACs. At higher concentrations, ACY-1215 inhibits HDAC 2, 3, 1, 8, 7, 5, 4, 9, 11 and SIRT 1/2 (Figure S4A,B) [40]. Thus, correlations between these HDAC isoforms and UM OS/PFS probability were analyzed (Figure S4C). *HDAC2* (Cox OS, *p* = 0.1; Cox PFS, *p* = 0.454), *HDAC3* (Cox OS, *p* = 0.443; Cox PFS, *p* = 0.293) and *HDAC1* (Cox OS, *p* = 0.219; Cox PFS, *p* = 0.408) expression does not correlate with OS or PFS, respectively. Intriguingly, high *HDAC11* expression correlated significantly with better OS and PFS (Cox OS, *p* = 0.006; Cox PFS, *p* = 0.024). On the other hand, *HDAC8* (Cox OS, *p* = 0.231), *HDAC7* (Cox OS, *p* = 0.751), *HDAC5* (Cox OS, *p* = 0.837), *HDAC4* (Cox OS, *p* = 0.34), *HDAC9* (Cox OS, *p* = 0.704) and *SIRT1* (Cox OS, *p* = 0.579) expression did not significantly correlate with overall survival probability. Low expression of *HDAC8* (Cox PFS, *p* = 0.024), *HDAC7* (Cox PFS, *p* = 0.05), *HDAC5* (Cox PFS, *p* = 0.012), *HDAC4* (Cox PFS, *p* = 0.012), *HDAC9* (Cox PFS, *p* = 0.00001) and *SIRT1* (Cox PFS, *p* = 0.023) significantly correlated with a better PFS probability. There was significant correlation between high *SIRT2* expression and OS probability (Cox OS, *p* = 0.025), while its expression did not correlate with PFS (Cox PFS, *p* = 0.531). In summary, HDAC6 expression levels were not altered across the three UM/MUM cell lines analyzed, and high *HDAC6* expression level was associated with better survival for UM patients.

### 2.4. Proteome Profiling Uncovers Molecular Signals Altered in OMM2.5 Cells by ACY-1215

**Figure 4:**
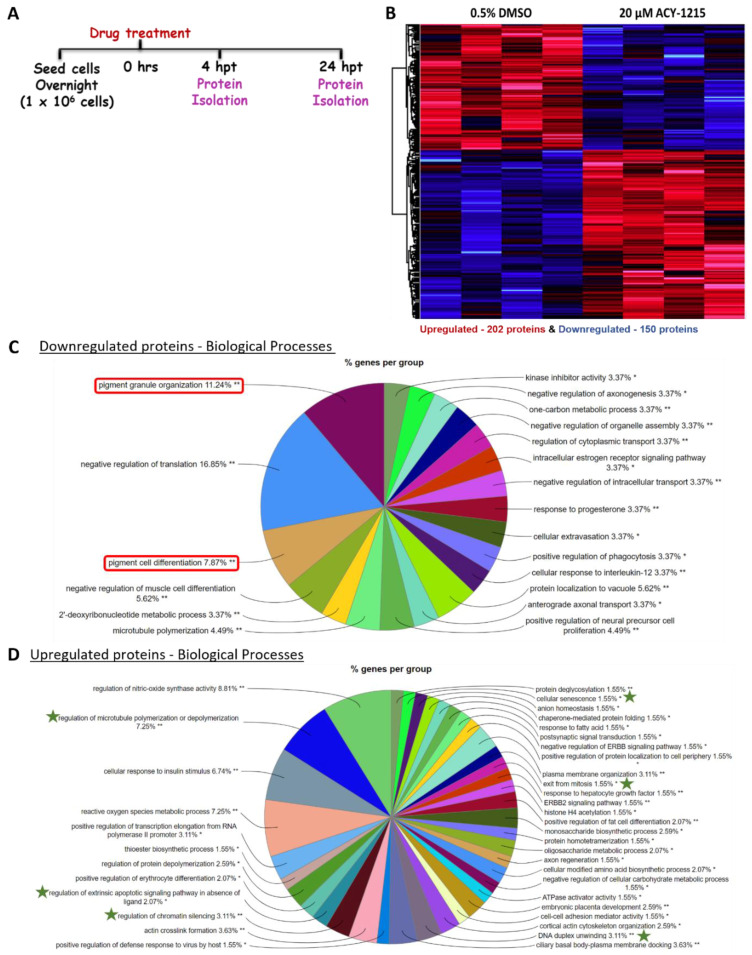
Proteome profiling of ACY-1215-treated cells to uncover mechanism of action. (**A**): ACY-1215 treatment regime for proteome profiling of OMM2.5 cells. Hours post treatment (hpt). (**B**): Heat map showing all significant differentially expressed proteins at 24 h post 20 μM ACY-1215 treatment in OMM2.5 cells. A total of 4423 proteins were identified in MS, with 150 downregulated (blue) and 202 upregulated (red) proteins (N = 4). (**C**,**D**): Enriched protein pathway analysis for GO term: biological processes for down and upregulated proteins, given a fold change cut off of +/− ≥ 1.2, *p* ≤ 0.05 displayed as pie charts. *, *p* < 0.05 and **, *p* < 0.01 denotes GO-term significance.

**Figure 5:**
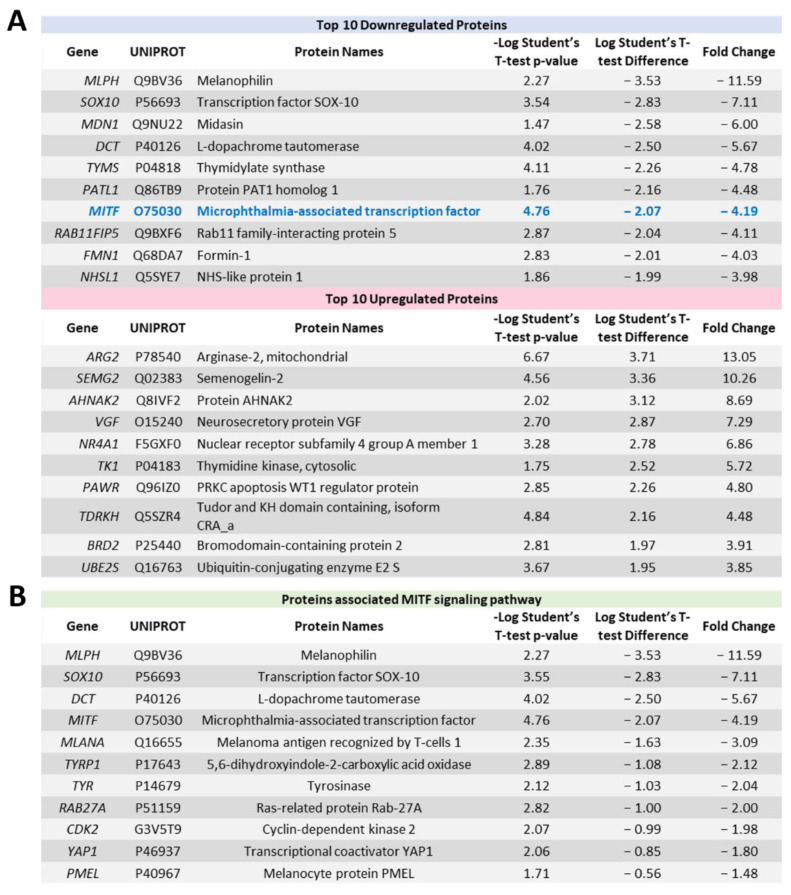
Significantly altered proteins identified by proteomic profiling following ACY-1215 treatment in OMM2.5 cells for 24 h. (**A**): Table highlighting the top 10 most down and upregulated proteins at 24 h post treatment (hpt) with 20 μM ACY-1215. (**B**): List of proteins involved in the MITF signaling pathway that were downregulated upon 20 μM ACY-1215 treatment for 24 h.

Having observed beneficial effects against the growth and viability of UM cell lines in vitro, proteome profiling of ACY-1215-treated OMM2.5 cells was performed to investigate the molecular mechanism of its anti-cancer action (Figure 4 and Figure S5, Tables S1 and S2). Changes in protein expression levels were analyzed after 4 and 24 h of 20 µM ACY-1215 treatment (Figure 4A). A total of 4,423 proteins were detected across all samples by mass spectrometry. At 4 hpt, 42 proteins were differentially expressed, with 11 proteins significantly upregulated and 30 proteins significantly downregulated (Figure S5A,B). Using the Cluego pathway analysis, the terms “dendrite development” and “regulation of G protein-coupled receptor signaling pathways” were identified as downregulated (Figure S5C). A distinct pathway was not detected within the upregulated proteins. At 24 hpt, 150 proteins and 202 proteins were significantly down and upregulated, respectively (Figure 4B). GO pathway enrichment analysis (fold change of > 1.2) for biological processes identified multiple pathways downregulated by ACY-1215, with pigment granule organization (11.24% of proteins) and pigment cell differentiation (7.87% of proteins) being prominently altered (Figure 4C and Figure S6A and Table S1). Through enriched pathway analysis, biological processes, such as regulation of microtubule polymerization or depolymerization (7.25% of proteins), DNA duplex unwinding (3.11% of proteins), regulation of chromatin silencing (3.11% of proteins), regulation of extrinsic apoptotic signaling pathway in absence of ligand (2.07% of proteins), cellular senescence (1.55% of proteins), exit from mitosis (1.55% of proteins) and ERBB2 signaling pathway (1.55% of proteins) were significantly upregulated by ACY-1215 treatment in OMM2.5 cells (Figure 4D and Figure S6B and Table S2). Proteins arginase-2, mitochondrial (ARG2; 13.05-fold), semenogelin-2 (SEMG2; 10.26-fold), protein AHNAK2 (AHNAK2; 8.69-fold), neurosecretory protein VGF (VGF; 7.29-fold), nuclear receptor subfamily 4 group A member 1 (NR4A1; 6.86-fold), thymidine kinase, cytosolic (TK1; 5.72-fold), PRKC apoptosis WT1 regulator protein (PAWR; 4.80-fold), Tudor and KH domain-containing protein (TDRKH; 4.48-fold), Bromodomain-containing protein 2 (BRD2; 3.91-fold) and ubiquitin-conjugating enzyme E2 S (UBE2S; 3.85-fold) were within the top ten significantly upregulated proteins (Figure 5A).

**Figure 6:**
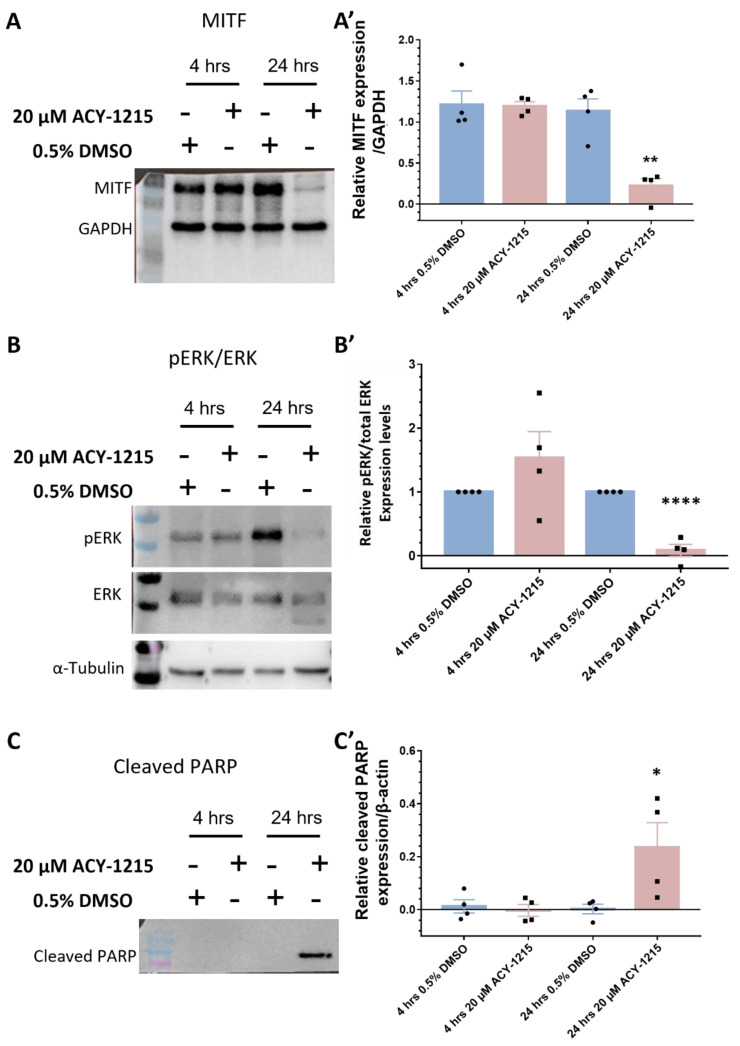
Western blot validation of proteomics data in OMM2.5 cells. (**A**,**A’**): There was no change in MITF expression levels after 4 h of 20 μM ACY-1215 treatment, while treatment for 24 h with 20 μM ACY-1215 led to a significant (**, *p* = 0.002) reduction in MITF expression levels. (**B,B’**): Relative expression levels of p-ERK to total ERK remained unchanged after 20 μM ACY-1215 treatment for 4 h. At 24 h post treatment with 20 μM ACY-1215, relative expression levels of p-ERK to total ERK were significantly (****, *p* < 0.0001) downregulated when compared to the 0.5% DMSO treatment. (**C**,**C’**): Expression of cleaved PARP was significantly (*, *p* = 0.049) upregulated after 24 h treatment with 20 μM ACY-1215 in comparison to 0.5% DMSO-treated OMM2.5 cells. Representative blots for each protein probed and densitometry analysis presented, raw blots are provided in Supplementary Figure S7. β-actin, GAPDH or α-tubulin were used as loading controls. Student’s Unpaired T test statistical analysis was performed, and data presented as mean ± SEM.

Interestingly, from the top 10 downregulated proteins, MITF was downregulated 4.19-fold by ACY-1215, with proteins connected to MITF signaling also strongly downregulated, i.e., melanophilin (MLPH; 11.59-fold), SRY-box transcription factor (SOX10; 7.11-fold) and L-dopachrome tautomerase (DCT; 5.67-fold), compared to vehicle control (Figure 5A). Corroborating our proteomics data, MITF expression was significantly downregulated in immunoblots (*p* = 0.002) following 24 h of 20 µM ACY-1215 treatment (Figure 6A,A’ and Figure S7B). A significant difference in MITF expression was not detected after 20 µM ACY-1215 treatment for only 4 h compared to vehicle control. Expression of additional MITF target proteins and regulators, such as melanoma antigen recognized by T cells 1 (MLANA; 3.09-fold), 5,6-dihydroxyindole-2-carboxylic acid oxidase (TYRP1; 2.12-fold), tyrosinase (TYR; 2.04-fold), Ras-related protein Rab-27A (RAB27A; 2.00-fold), cyclin-dependent kinase 2 (CDK2; 1.98-fold), transcriptional coactivator YAP1 (YAP1; 1.80-fold), melanosome protein PMEL (PMEL; 1.48-fold), were significantly reduced by ACY-1215 (Figure 5B). Furthermore, phospho-ERK and ERK expression levels were analyzed in order to determine whether the mitogen-activated protein kinase (MAPK)/ERK signaling pathway played a role in the ACY-1215 mechanism of action. Through immunoblotting, a significant difference in p-ERK expression levels was not observed after 4 h of 20 µM ACY-1215 treatment compared to vehicle control (Figure 6B,B’ and Figure S7C). Following 24 hpt with 20 µM ACY-1215, relative p-ERK expression levels were significantly downregulated (*p* <0.0001) compared to vehicle control (Figure 6B and Figure S7C). Overall, through proteomic analysis, reduced MITF and p-ERK levels were linked to ACY-1215 treatment of OMM2.5 cells.

### 2.5. ACY-1215 Treatment Arrests OMM2.5 Cell Cycle Progression in S Phase

**Figure 7:**
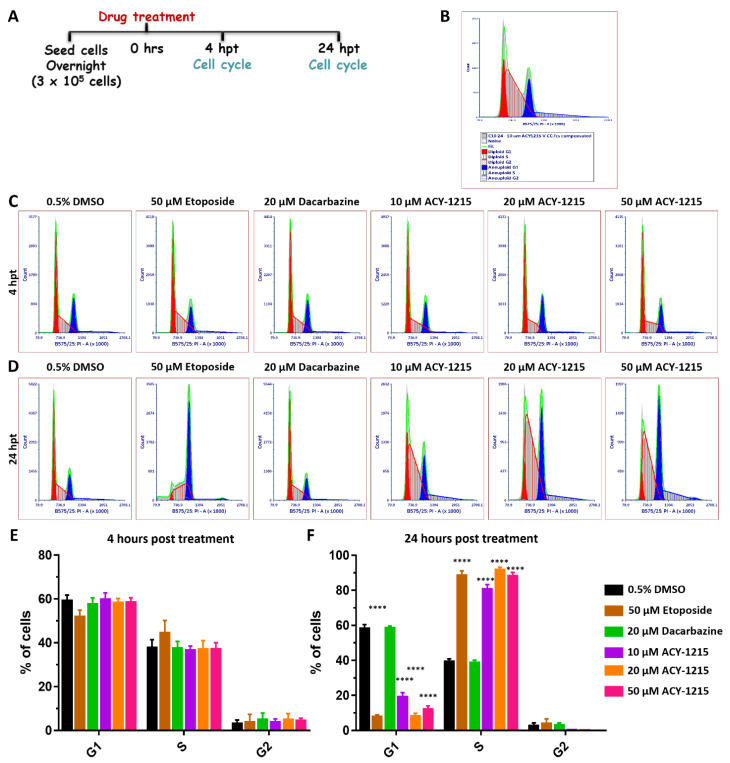
Cell cycle progression is arrested in the S phase by ACY-1215 in OMM2.5 cells. (**A**): Schematic illustrating treatment protocol undertaken. Hours post treatment (hpt). (**B**): Flow cytometry data analysis plot legend. (**C**,**E**): 4 h of treatment with 10, 20 or 50 μM ACY-1215, or 50 μM Etoposide or 20 μM Dacarbazine did not alter the cell cycle profile. (**D**,**F**): A significant (****, *p* = 0.0001) reduction in the percentage of cells in G_1_ phase and a significant (****, *p* = 0.0001) increase in the percentage of cells in the S phase were observed after 24 hpt with 10, 20 or 50 μM ACY-1215 or 50 μM Etoposide, in comparison to vehicle control. No changes in the cell cycle phases were observed following 20 μM Dacarbazine treatment. No alterations to G_2_ phase were observed in all treatment groups. Statistical analysis was performed by two-way ANOVA with Dunnett’s Test for Multiple Comparisons and data represented as mean ± SEM (*N* = 4).

Outside of UM, previous studies have independently demonstrated that ACY-1215 and MITF regulate the cell cycle [41,42,43,44,45]. To determine whether ACY-1215 treatment altered cell cycle phases, OMM2.5 cells were treated with either 0.5% DMSO, 10, 20 or 50 µM of ACY-1215, 50 µM Etoposide (a chemotherapeutic used as a positive control for apoptotic cell death) or 20 µM Dacarbazine for 4 and 24 h. The cells were isolated, fixed, labeled with propidium iodide, and analyzed using flow cytometry (Figure 7 and Figure S8). In line with published studies, OMM2.5 cells underwent two cell cycle phases, due to the DNA ploidy of UM cells [46,47]. Approximately 60–70% of the cell population were diploid, in cell cycle 1, and the remaining cell population presented with aneuploidy in cell cycle 2 (Figure S8). Significant changes in G_1_, S and G_2_ cell cycle phases were not observed after 4 h of ACY-1215 in any treatment group compared to vehicle controls (Figure 7B,C,E). After 24 h of treatment with Etoposide or ACY-1215, a significant reduction (*p* = 0.0001) in the number of cells in G_1_ phase and a significant increase (*p* = 0.0001) in the number of cells in S phase were identified across the treatment groups compared to vehicle controls (Figure 7B,D,F). On average, 19.0%, 8.2% and 11.9% of OMM2.5 cells were in G_1_ phase following 10, 20 and 50 µM of ACY-1215 treatment, respectively, compared to 58.0% of cells in G_1_ when treated with vehicle control. On average, 80.7%, 91.6% and 88.0% of cells were detected in the S phase upon treatment with 10, 20 and 50 µM of ACY-1215 in comparison to 39.35% of 0.5% DMSO-treated cells. OMM2.5 cells treated with 20 µM Etoposide had 7.8% of cells in G_1_ and 88.4% of cells in the S phase. The number of cells in G_2_ phase across all treatment groups did not significantly change at 24 hpt. No change was observed in any of the cell cycle phases following Dacarbazine treatment at 4 or 24 h. In summary, cell cycle analysis proved that ACY-1215 treatment for 24 h attenuated OMM2.5 cell cycle progression in the S phase.

### 2.6. Elevated Apoptosis Results from ACY-1215 Treatment of OMM2.5 Cells

**Figure 8:**
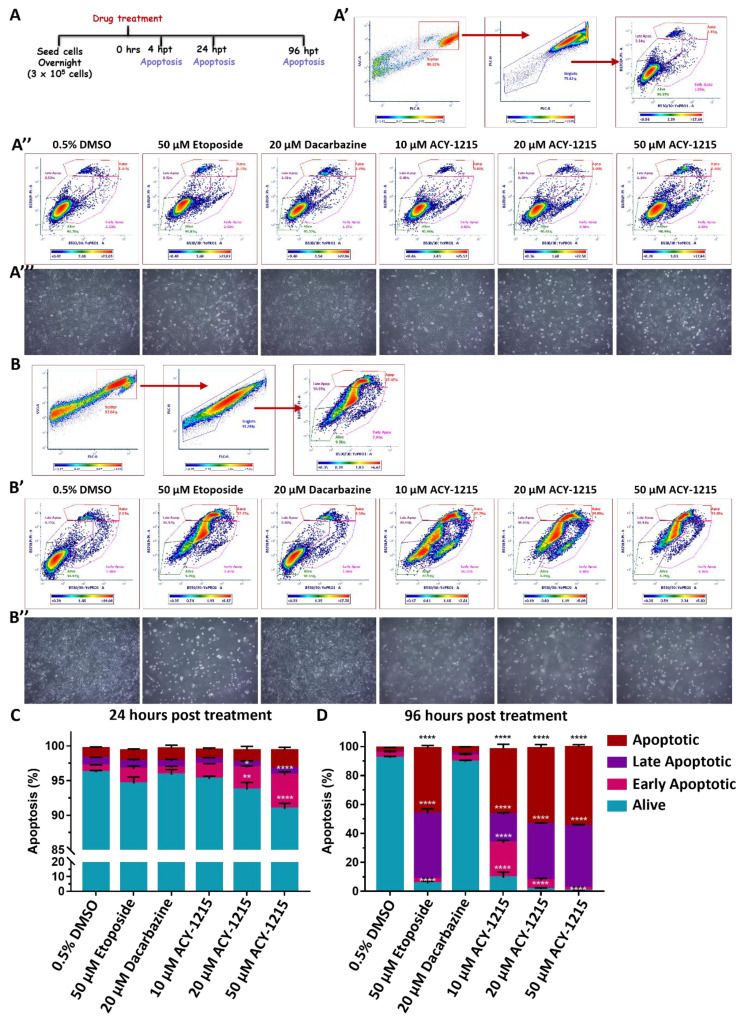
ACY-1215 activates the apoptotic pathway in OMM2.5 cells. (**A**): Diagram portraying treatment regime. Hours post treatment (hpt). (**A’**,**B**): Plots representing gating of cell singlets into different stages of apoptosis. (**A’’**,**B’**): Representative plots depicting OMM2.5 treated with 0.5% DMSO; 10, 20 or 50 μM ACY-1215; 50 μM Etoposide or 20 μM Dacarbazine, at 24 and 96 hpt, respectively. (**A’’’**,**B’’**): Representative micrographs of OMM2.5 cells at 24 and 96 h post treatment. (**C**): A significant reduction in the percentage of live cells and a significant increase in the percentage of early apoptotic cells was detected following 20 μM (**, *p* = 0.005 and *, *p* = 0.017, respectively) and 50 μM (****, *p* < 0.0001) ACY-1215 treatment compared to 0.5% DMSO treatment. (**D**): Live cell populations were significantly (****, *p* < 0.0001) reduced, and cell populations in late apoptotic stage and apoptotic stage were significantly (****, *p* < 0.0001) increased upon 50 μM Etoposide and all concentrations of ACY-1215 tested. The 10 μM ACY-1215 treatment resulted in a significant increase in the early apoptotic cell population compared to 0.5% DMSO. The 20 μM Dacarbazine treatment was comparable to vehicle control plots. Statistical analysis by two-way ANOVA, followed by Tukey’s Multiple Comparisons test, with error bars shown as mean ± SEM (N = 3).

As the majority of OMM2.5 cells were arrested at the S phase after 24 h of ACY-1215 treatment, we investigated whether these cells underwent increased apoptosis. OMM2.5 cells were treated, isolated, labeled with YO-PRO^TM^-1 Iodide and Propidium iodide to distinguish between viable, non-viable and cells in different apoptotic stages (Figure 8 and Figure S9). In line with our cell cycle results, 4 h of ACY-1215 treatment did not significantly alter apoptotic cell number in any treatment group (Figure S9). At 24 hpt, a significant reduction in live cells was reported with 20 µM (2.52% reduction of total number of live cells; *p* = 0.0055) and 50 µM (5.28% reduction of total number of live cells; *p* < 0.0001) ACY-1215 compared to the vehicle control (Figure 8A’,A’’,C). Additionally, ACY-1215 significantly increased the average number of early apoptotic cells, as evidenced by 3.22% (*p* = 0.017) and 4.89% (*p* < 0.0001) early apoptotic cells following 20 µM or 50 µM ACY-1215 treatment, respectively, compared to the vehicle control. After 24 h of treatment, there was no significant difference detected in the average number of cells undergoing late apoptosis or dead cells across all treatment groups (Figure 8A’’,C). In line with our findings, cleaved PARP expression (a marker for apoptosis) was significantly upregulated at 24 hpt with 20 µM ACY-1215 (*p* = 0.049) and not at 4 hpt (Figure 6C,C’ and Figure S7D).

Prolonged ACY-1215 treatment for 96 h resulted in the majority of cells being either non-viable or undergoing late apoptosis (Figure 8B,B’,D). The average number of viable cells with 10, 20 or 50 µM ACY-1215 was significantly reduced to 9.47% (*p* < 0.0001), 1.56% (*p* < 0.0001) and 0.46% (*p* < 0.0001), respectively. In contrast, 92.9% and 89.45% of cells were viable in vehicle control- and 20 µM Dacarbazine-treated groups, on average, respectively (Figure 8B’,D). A significant increase in early apoptotic cells was detected in the 10 µM ACY-1215 treatment with 23.95% (*p* < 0.0001) of cells, compared to the vehicle control; a significant change was not observed in all other treatment groups. 1.15% of cells in the 0.5% DMSO treatment group and 1.36% of cells treated with 20 µM Dacarbazine were in late apoptotic stage, while a substantial number of cells, on average 19.8% (*p* < 0.0001) in 10 µM, 39.0% (*p* < 0.0001) in 20 µM and 42.9% (*p* < 0.0001) in 50 µM ACY-1215-treated groups, were undergoing late-stage apoptosis (Figure 8B’,D). ACY-1215 treatment resulted in a profound number of non-viable cells in a dose-dependent manner, with 44.8% (*p* < 0.0001), 52.4% (*p* < 0.0001) and 54.5% (*p* < 0.0001) following 10, 20 and 50 µM concentrations, in comparison to 2.71% dead cells in vehicle control- and 4.47% in 20 µM Dacarbazine-treated groups (Figure 8B’,D). Etoposide (50 µM), a positive control for apoptosis, showed 5.41% (*p* < 0.0001) cells were viable, 45.2% (*p* < 0.0001) were in late apoptotic stage and 45.0% (*p* < 0.0001) were non-viable (Figure 8B’,D). Furthermore, micrograph images of all treated cells corroborate our results that 96 h of treatment with Etoposide or ACY-1215 significantly reduced cell viability, with most of the cells not adhering to the culture plate, in contrast to the vehicle control or clinical chemotherapeutic for 24 h treatment groups (Figure 8A’’’,B’’). Overall, we observe a time- and dose-dependent alteration in OMM2.5 cell viability, cell cycle arrest and triggering of apoptosis, 24 h post ACY-1215 treatment.

### 2.7. MITF Inhibitor Treatment Prevents OMM2.5 Cell Survival and Proliferation In Vitro

**Figure 9:**
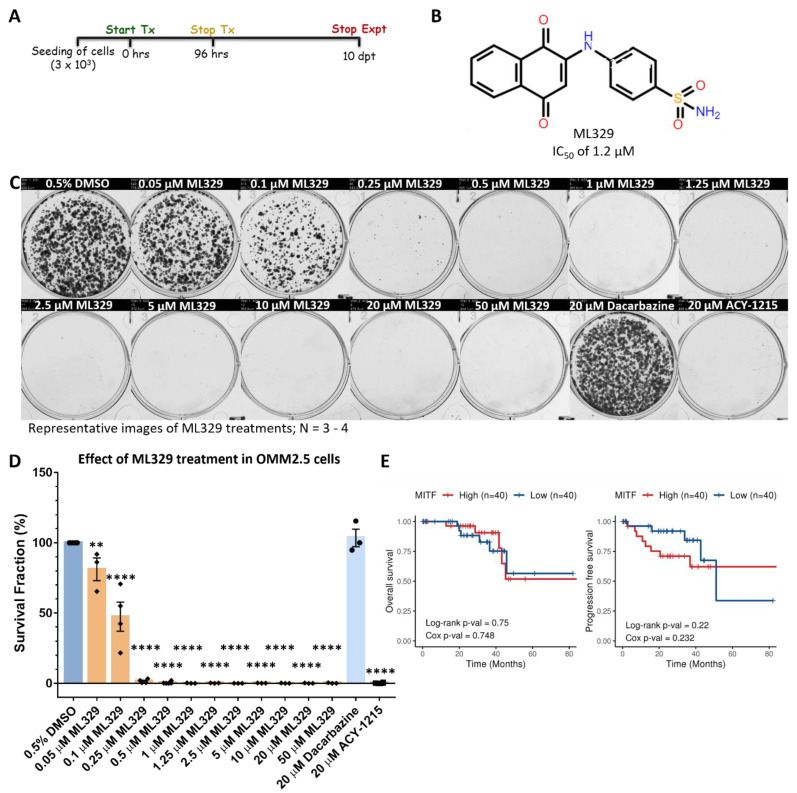
Inhibition of MITF pathway reduces OMM2.5 cell proliferation in vitro. (**A**): Schematic diagram of treatment regime. Days post treatment (dpt). (**B**): Chemical structure of ML329, a small molecule MITF pathway inhibitor. (**C**): Representative image of clonogenic assay plates for OMM2.5 cells treated with 0.5% DMSO, 0.05–50 μM ML329, 20 μM Dacarbazine or 20 μM ACY-1215 for 96 h. (**D**): A dose-dependent, significant reduction in the number of OMM2.5 colonies was observed in ML329 treatment groups compared to the 0.5% DMSO treatment group. One-way ANOVA with Dunnett’s Test for Multiple Comparisons statistical analysis was performed; error bars represent mean ± SEM, **, *p* = 0.005, ****, *p* = 0.0001 (*N* = 3–4). (**E**): Kaplan–Meier survival curves demonstrating no correlation between expression of MITF and overall survival (OS) or progression-free survival (PFS) in UM patients. Median values were used as cut-off for high (red) and low (blue) expression levels, with Log-rank *p*-values (categorical variable) and Cox *p*-values (continuous variable) calculated (*n* = 80).

To further interrogate the requirement of MITF in OMM2.5 cell survival, cells were treated with the MITF pathway inhibitor ML329, and survival and proliferation was analyzed using colony formation assays. Cells were treated with increasing doses of ML329, ranging between 0.05 µM and 50 µM, given the reported IC_50_ value of 1.2 µM (TRPM-1 promoter assay) (Figure 9A,B) [48]. The treatment regime was performed as previously described, whereby OMM2.5 cells were treated with respective drug doses for 96 h and then maintained in culture, in fresh complete media for an additional 10 days (Figure 9A).

ML329 induced a significant reduction in the average number of surviving OMM2.5 colonies (reduced by 18.9%, *p* = 0.005) when treated with 0.05 µM ML329 treatment compared to 0.5% DMSO (Figure 9C,D). At higher concentrations of ML329, more pronounced effects were detected, with significant reductions in viable clones averaging 52.6% to 99.8% (*p* = 0.0001) decreases at 0.1 to 50 µM concentration of ML329, compared to vehicle controls (Figure 9C,D). Corroborating our data, the treatment of OMM2.5 cells with 20 µM Dacarbazine did not result in a significant difference in the average number of viable clones, while 20 µM ACY-1215 treatment led to a significant reduction (99.8%; *p* = 0.0001) in the number of surviving clones, compared to 0.5% DMSO. Given that MITF was found to play a role in OMM2.5 cell survival, correlations between *MITF* expression and UM patient OS/PFS was investigated. Curiously, high or low *MITF* expression levels were not significantly associated with better OS nor PFS (Cox OS, *p* = 0.748 and Cox PFS, *p* = 0.232), as shown by the Kaplan–Meier survival curves (Figure 9E).

### 2.8. Inhibition of MITF Pathway Reduces OMM2.5 Cell Fluorescence In Vivo in Zebrafish Xenograft Models

**Figure 10:**
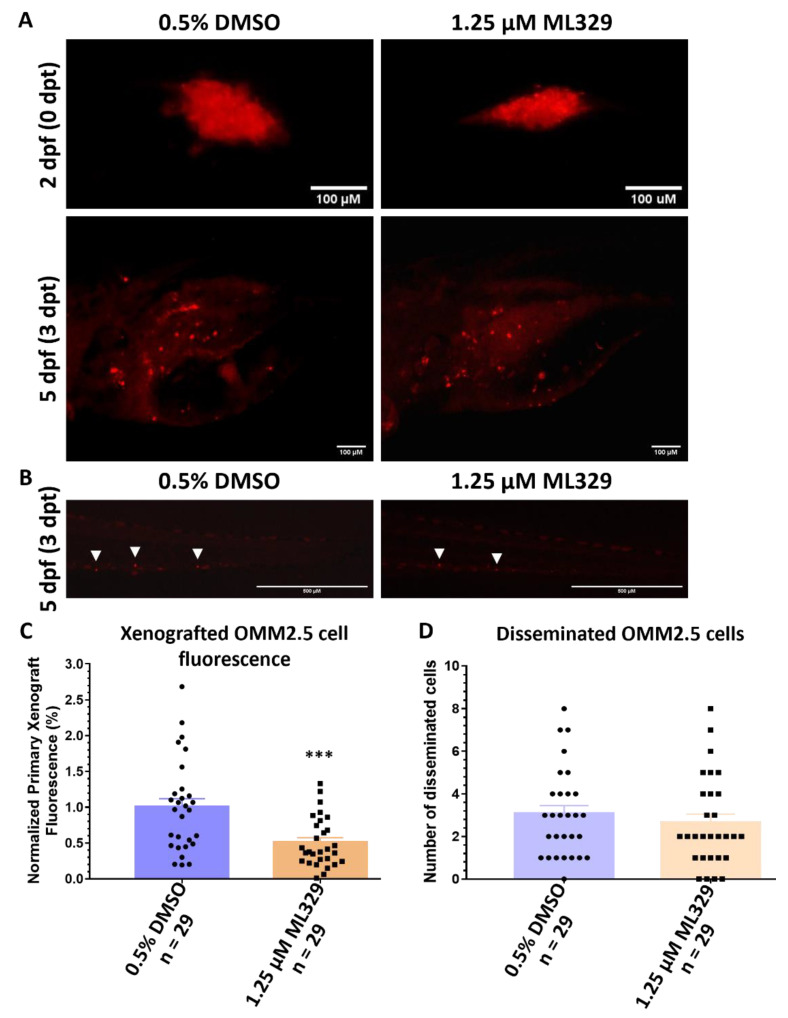
ML329 demonstrates anti-UM properties in zebrafish OMM2.5 xenografts. (**A**): Top panel shows OMM2.5 Dil-labeled cells xenografted into the perivitelline space of 2-day-old zebrafish larvae. Bottom panel presents zebrafish larvae 3 days post treatment (dpt) with 0.5% DMSO (*n* = 29) or 1.25 μM ML329 (*n* = 29). Days post fertilization (dpf). (**B**). Representative image of OMM2.5 Dil-labeled cells disseminated (white arrowhead) to the caudal vein plexus of zebrafish larvae at 3 dpt. (**C**): A significant (***, *p* = 0.0006) regression of the average normalized primary xenograft fluorescence of OMM2.5 Dil-labeled cells was observed when treated with 1.25 μM ML329. (**D**): No difference was detected in the average number of disseminated OMM2.5 Dil cells following treatment with 1.25 μM ML329 compared to vehicle control. Student’s T test was used for statistical analysis, with error bars presenting mean ± SEM.

The effect of the MITF pathway inhibitor ML329 on the Dil-labeled fluorescent signal of OMM2.5 cells was determined in vivo, using zebrafish xenograft models. ML329 was well tolerated by zebrafish in vivo, albeit with drug precipitation at higher concentrations (1–100 µM) (Figure S10). Although we observed effects in vitro at concentrations as low as 0.25 µM ML329, we chose the concentration of 1.25 µM for our study to fit with the reported IC_50_ value [48]. As before, OMM2.5 Dil-labeled cells were injected into the perivitelline space, after which the larvae (2 dpf) were treated with either 0.5% DMSO or 1.25 µM ML329 for 3 days (Figure 10A). There was no significant difference in the average number of disseminated cells to the caudal vein plexus of the OMM2.5 xenografted larvae at 0.5% DMSO (3.1 cells) or 1.25 µM ML329 (2.6 cells) treatment groups (Figure 10B,D). However, on average, a 49% (*p* = 0.0006) reduction in OMM2.5 primary xenograft cell fluorescence was detected after normalization, following treatment with 1.25 µM ML329 compared to vehicle controls (Figure 10A,C). Experimentally, therefore, we observe a beneficial effect of blocking the MITF pathway in the OMM2.5 cell line in vitro and in vivo.

## 3. Discussion

Metastatic UM (MUM) is a poor prognosis cancer, lacking effective treatment options. Our study has provided evidence that small molecule drugs ACY-1215 and ML329 are efficacious in conferring anti-cancer effects in a MUM cell line, both in vitro and in vivo. To the best of our knowledge, this is the first study to provide evidence linking the HDACi ACY-1215 and MITF in OMM2.5 MUM cells.

Three commercially available, first-generation HDAC6i were screened in UM and MUM cell lines, and ACY-1215 was selected for follow-up studies. ACY-1215, either as a monotherapy or in combination with other drugs, is presently in clinical trials for several cancers [27,49]. We observed strong anti-cancer effects elicited by ACY-1215 treatment in a dose-dependent manner in both UM- and MUM-derived cell lines, albeit weak HDAC6 expression is reported in UM tissues [50]. Notably, HDAC6 activity is significantly increased in inflammatory breast cancer, even though HDAC6 is not overexpressed [51]. Hence, it is plausible that in MUM, there is increased HDAC6 activity, but not HDAC6 expression. Our data indirectly support the findings by Nencetti et al., whereby a novel synthetized quinoline derivative VS13, with high selectivity against HDAC6, led to a reduction in UM cell viability in vitro [26]. In addition, here, the anti-cancer effect of ACY-1215 on the transplanted OMM2.5 cell mass was demonstrated in vivo in zebrafish OMM2.5 xenograft models, without any significant change to the number of disseminated cells. This is not surprising, given the timeframe of the experiment and a low burden in the average number of disseminated cells to the caudal vein plexus three days post transplantation in the vehicle controls. It would be worthwhile to perform follow-up studies to evaluate the efficacy of ACY-1215 on tumor metastasis, with long-term treatment regimens and in different MUM tumor-derived cell lines, patient-derived samples in vivo in larvae and/or in adult zebrafish [52,53,54,55].

However, pure HDAC6 inhibition mediated effects must be inferred with caution, as higher doses of ACY-1215 result in non-selective inhibition, and the observed beneficial effects are mediated by additional targets [40,56]. In a study by Lin et al., CRISPR-induced HDAC6 knock-out lines (e.g., melanoma, triple negative breast cancer, colorectal cell lines) demonstrated that the cell viability/proliferation capability was comparable to wildtype controls; additionally, ACY-1215 was able to mediate its anti-cancer effects at high concentrations (micromolar), even when HDAC6 was knocked-out [56]. Corroborating their findings, Depetter et al. revealed that treatment with 10 μM ACY-1215 in HAP1 cells with HDAC6 knock-out led to a reduction in cell viability [40]. In another study, a distinct anti-proliferative effect was observed in high-grade serous ovarian cancer cells when a non-selective concentration of 10 μM ACY-1215 was used [57]. In both studies, the authors suggested that the true beneficial effects of HDAC6 inhibition might be reaped in combinatorial therapy rather than when administered as a single agent. Therefore, it is acknowledged that at the treatment concentration of 20 μM, we are potentially non-selectively targeting other factors, such as Class I HDAC isozymes, given the reported IC_50_ value for ACY-1215 in enzymatic assays is 4.7 nM. In OMM2.5 cells, we do not observe a significant reduction in cell survival and viability at ACY-1215 treatment concentrations of less than 5 μM; therefore, we postulate that using lower concentrations of ACY-1215 that are within the selective range for HDAC6 inhibition will not offer the desired treatment benefits in this cell line. Importantly, HDAC6 was indeed inhibited by ACY-1215 at the concentration we used, as its substrate, acetylated α-tubulin, was significantly upregulated. Furthermore, from our proteomics data we also identified proteins involved in microtubule polymerization and regulation of microtubule polymerization or depolymerization to be significantly altered [58]. Irrespective of non-selective inhibition of HDAC isozymes, ACY-1215 still presents as a promising therapeutic option for treatment of MUM, with its ability to prevent UM cell growth, that warrants further interrogation.

Proteome profiling of ACY-1215-treated OMM2.5 cells was key in deducing potential mechanisms of action. We discovered that the MITF signaling pathway and associated factors were significantly downregulated upon treatment with 20 μM ACY-1215, given our treatment regime. Tying in with the concentration of ACY-1215 used, our findings are in line with another study, whereby it was reported that treatment of melanoma and clear cell sarcoma cells with different pan-HDAC inhibitors resulted in reduced MITF expression in vitro and in vivo in a mouse melanoma xenograft model [59].

The role of MITF has been extensively studied in cutaneous melanoma [60,61,62]. MITF is a key transcription factor and a master regulator of melanogenesis and melanocyte differentiation. It also plays a multifaceted role regulating several cellular processes, including cell cycle, DNA damage repair, lysosome biogenesis, metabolism, autophagy and oxidative stress [63,64,65,66]. MITF can be further distinguished into five different isoforms: MITF-A, MITF-B, MITF-C, MITF-H and MITF-M [67]. In particular, in cutaneous melanoma, MITF-M is involved in carcinogenesis events, such as survival, proliferation, differentiation, invasion and migration [62]. Unsurprisingly, certain types of mutations in MITF and MITF-associated members are linked to oncogenic functions in melanoma [63,68,69]. MITF plays a dual role in cutaneous melanoma, based on its expression levels and activity; however, there is controversy surrounding this matter [64]. For instance, some studies report that low MITF expression is necessary for proliferation, and higher levels of MITF correlate with suppression of cell proliferation and promote differentiation [62]. Meanwhile, others state that low levels of MITF expression are linked to invasiveness, whereas high levels of MITF expression are required for cell proliferation/differentiation [43,61,70]. Nevertheless, targeting the MITF pathway shows promise as an anti-cancer approach. Aida et al. demonstrated that the growth of melanoma cells, SK-MEL-5 and SK-MEL-30, were inhibited by siRNA-mediated knock-down of MITF [71]. Similarly, in another study, a knock-down of MITF by shRNA in MM649 cells resulted in reduced cell proliferation in vitro and tumor growth and dissemination in vivo in mouse xenografts [60]. Furthermore, pharmacological inhibition of the MITF signaling pathway using small molecule ML329 reduced cell viability in MITF-dependent melanoma (SK-MEL-5 and MALME-3M) cells without affecting the viability of A375 cells, a MITF-independent cell line [48]. Comparably, another compound, CH5552074, inhibited the growth of SK-MEL-5 cells via the suppression of MITF protein [71]. Interestingly, a knock-down of MITF in B16F10 melanoma cells and overexpression of MITF in YUMM1.1 cells led to increased tumor growth in vivo in mice [72]. Apart from melanoma, studies have connected MITF with a role in multiple cancers, including non-small cell lung cancer, pancreatic cancer and hepatocellular carcinoma [73,74,75]. Most recently, it was demonstrated that a knockdown of MITF in clear cell renal cell carcinoma cells resulted in reduced cell proliferation and an increase in cells in S/G_2_ phases, suppressed cell migration and invasion in vitro and tumor formation in vivo; an opposite effect was observed when MITF was overexpressed [44]. In the context of UM, MITF is upregulated in UM cells [76]. In our study, expression levels of MITF and several proteins involved in pathways associated with MITF, such as pigment cell differentiation and melanosome organization, were downregulated upon ACY-1215 treatment of OMM2.5 cells. This was consistent with the observed trend when MITF is downregulated. Taken together, this suggests that targeting the MITF signaling pathway may have therapeutic value in MUM that needs to be explored in-depth. Further interrogation of the mechanism of action via immunoprecipitation or co-immunoprecipitation could uncover a direct link between MITF and HDAC isozymes. Future studies could screen MITF/MITF pathway inhibitors in additional primary (e.g., 92.1, Mel270, Mel290, SP-6.5) and liver/skin metastatic (e.g., OMM1, MM28, MM33, MM66,) UM cell lines in vitro or in vivo. Tumor tissue samples collected from UM/MUM patients could be assessed ex vivo or in vivo in xenograft models. Data obtained from these assays will provide a broader insight into the potential therapeutic benefits of targeting the MITF pathway for UM/MUM, increase our understanding of the molecular mechanisms involved, as well as ascertain the implications with regard to drug response and genetic background, opening up avenues for targeted therapy.

Significantly, several studies have independently shown that ACY-1215 regulates cell cycle and cell death mechanisms in various cancers. In HCT-116 and HT29 colorectal cancer cells, a reduction in cell proliferation and viability was noted in a time- and dose-dependent manner, and apoptosis was also observed at non-selective ACY-1215 concentrations [42,77]. Interestingly, ACY-1215, when used at HDAC6 selective concentrations (up to 2 μM), did not promote apoptosis; however, if used in combination with other anti-cancer drugs, it proved to be more effective [77,78]. In esophageal squamous cell carcinoma cell lines (EC109 and TE-1), ACY-1215 treatment resulted in suppression of cell proliferation through the arrest of cell cycle in G_2_/M phase and an increase in apoptosis [79]. Similarly, 4 μM ACY-1215 treatment for 24 h prompted an increase in percentage of cells in G_0_/G_1_ phase and a time-/dose-dependent proapoptotic effects of ACY-1215 uncovered in lymphoma cell lines [41]. More recently, in gall bladder cancer cells, ACY-1215 inhibited cell proliferation and induced apoptosis, as well as enhancing the chemotherapeutic effects of other anti-cancer agents upon co-treatment [80]. Collectively, in these studies it became evident that the PI3K/AKT and MAPK/ERK pathways played a central role in ACY-1215 mechanism of action. We postulated whether ACY-1215 treatment promoted cell cycle arrest and apoptosis in OMM2.5 cells. As expected, at the non-selective concentration, ACY-1215 treatment resulted in the halting of cell cycle progression in the S phase and induced apoptosis. We observed a significant increase in early apoptotic cells and a significant reduction in the number of viable OMM2.5 cells at 20 and 50 μM ACY-1215 treatment by 24 h. Additionally, the expression of cleaved PARP, which is used as an indicator for apoptosis, was markedly upregulated in ACY-1215-treated OMM2.5 cells at 24 h post treatment [81,82]. Further supporting evidence can be drawn from our proteomics data, whereby the pathways—regulation of extrinsic apoptotic signaling pathway in absence of ligand, exit from mitosis and cellular senescence—were upregulated, indicating an increase in expression levels of proteins associated with these biological processes. By 96 h, at all tested ACY-1215 concentrations, the majority of cells were either apoptotic or in late apoptotic stages. Considering that MITF was significantly downregulated at 24 h post treatment by ACY-1215, the cause of increased cell death observed following ACY-1215 treatment is potentially mediated through the downregulation of MITF. In order to further confirm that the observed anti-cancer effects of ACY-1215 result from the regulation of MITF, OMM2.5 cells were treated with a MITF pathway inhibitor, ML329, in vitro and in vivo in zebrafish OMM2.5 xenografts. We noted a dose-dependent reduction in cell viability in vitro and inhibition of the MITF pathway at the tested concentration, revealed an anti-UM effect in vivo. Additional interrogation of the link between Class I/II HDAC isozymes and MITF opened up the possibility of MAPK/ERK signaling being linked to the ACY-1215 mechanism of action in OMM2.5 cells. We observed that p-ERK expression levels are significantly reduced following 24 h of ACY-1215 treatment. In other contexts, MAPK/ERK signaling pathway is involved in the ACY-1215 mechanism of action, and ERK1/2 and HDAC6 are interacting partners involved in a positive feed-forward loop [83,84,85]. In colon cancer cell lines, a knock-down of HDAC6 resulted in reduced p-ERK expression, but not total ERK expression levels [86]. In A375 melanoma cells, ACY-1215 alone and in combination with vemurafenib led to inactivation of ERK [87]. Interestingly, in prostate cancer cells (LNCaP), blocking of HDAC6 with Panobinostat led to increased ERK activity and, as a consequence, promoted apoptosis [88]. However, this was not the case in PC-3 prostate cancer cells. In another study, increased HDAC6 expression in lung cancer cell by Isoproterenol treatment led to inhibition of ERK signaling [89]. This indicates cell-specific HDAC-ERK1 regulation and activity. In our study, we cannot elucidate whether increased ERK activity or expression levels mediate the observed reduction in MITF expression levels. In UM, *GNAQ/GNA11* mutations are associated with constitutive activation of the MAPK/ERK signaling pathway, although heterogeneity in MAPK/ERK signaling has been observed across UM samples with *GNAQ/GNA11* mutations [90,91,92,93]. More specifically, the Mel270 and OMM2.5 cells used in this study carry a mutation in *GNAQ*, which is known to result in constitutively active MAPK/ERK signaling in UM [8,94]. Reportedly, ~83% of UM cases harbor somatic mutations in GNAQ/GNA11, with ~46% of cases attributed to GNAQ^Q209^ mutations; hence, these cell lines represent a large cohort of UM tumors [95,96].

Going forward, it will be worthwhile to evaluate potential synergistic effects between ACY-1215 and Dacarbazine or MITF/MEK inhibitors in MUM cells, given that many studies are reporting increased benefits with combinatorial treatment regimes. Although promising, the role of ACY-1215 and ML329 needs to be thoroughly investigated in UM and MUM patient samples. Currently, there is no clear evidence linking either HDAC6/Class I/II HDAC or MITF in MUM prognosis. Immunohistochemistry-based expression analysis of 16 primary UM samples detected variable low levels of HDAC6 expression, with no correlation between HDAC6 expression levels and UM in this limited sample size [50]. Based on TCGA data analysis of 80 UM patient samples, a significant correlation was found between *HDAC6* expression and OS probability, highlighting a possible involvement of HDAC6 in UM prognosis. Moreover, *HDAC2* and *SIRT2* expression correlated with OS, while *HDAC4* expression showed correlations to PFS. HDAC 1 and 3 expression was not correlated with either OS or PFS, even though HDAC 1, 2, 3, 4 and Sirtuin 2 (SIRT2) expression was detected in UM eye samples [50]. A limited number of studies have explored the expression of MITF in UM and MUM. MITF expression was found in 100% (15 out of 15) of UM samples in one study; however, in another study, MITF expression was detected in 65% (37 out of 57 samples) of choroidal UM patient samples, with levels of MITF expression not significantly associated with the survival of these patients [97,98]. Comparably, from our TCGA data analysis, there was no correlation between MITF transcript expression levels and OS/PFS seen in UM patients. It has also been previously suggested that MITF would be a useful marker for ocular malignant melanoma [99]. Taken together, it will be worthwhile to perform an extensive study with a larger cohort of UM and MUM patient samples to conclusively determine whether MITF plays a part in MUM prognosis. However, one needs to be aware that expression data assess a different biological parameter compared to pharmacological effects. There is no a priori reason why MITF expression levels have to be upregulated in order for therapeutic effects of MITF inhibition to be observed. Additionally, it needs to be determined whether ACY-1215 and ML329 are effective, irrespective of the MUM causative mutation(s).

This study highlights that ACY-1215 regulates the survival of primary and metastatic UM cell lines and provides evidence that this involves the regulation of MITF in OMM2.5 cells. Our data suggest that ACY-1215- and ML329-related signaling pathways offer novel options for identifying therapeutic targets for the treatment of MUM, which needs to be considered and further evaluated.

## 4. Methods

### 4.1. Cell Culture

Mel270, Mel285 and OMM2.5 cells (kindly provided by Dr. Martine Jager, Leiden, The Netherlands) were cultured with complete media containing RPMI 1640 with GlutaMAX™ Supplement (Gibco; Waltham, MA, United States) + 10% fetal bovine serum (FBS; Gibco) + 2% penicillin–streptomycin (PEST) in T175 flasks for no more than 20 passages [35]. ARPE-19 cells were maintained in complete media containing DMEM: F12 supplement (Lonza; Basel, Switzerland) + 10% FBS + 1% PEST + 2.5 mM L-glutamine. Culture flasks and plates were incubated at 37 °C with 5% CO_2_.

### 4.2. Clonogenic Assay

All three cell lines were seeded into 6-well plates at 1.5 × 10^3^ cells/mL (final volume 2 mL) and allowed to adhere overnight. Initial drug screens were performed with Mel285 cells seeded at 1.5 × 10^3^ cells/mL, and Mel270/OMM2.5 cells were seeded at 9 × 10^3^ cells/mL. The following day, cells were treated with either 0.5% DMSO (vehicle control) or 20 µM Dacarbazine (clinical control; Sigma-Aldrich; St. Louis, MO, United States) or HDACi (Tubastatin A (SelleckChem; Houston, TX, USA), ACY-1215 (SelleckChem) and Tubacin (Sigma-Aldrich)) at increasing doses, ranging from 1 µM, 5 µM, 10 µM, 20 µM and 50 µM, prepared in complete media. MITF inhibitor ML329 (Ambeed, Inc.; Sigma-Aldrich) was tested at increasing concentrations, ranging from 0.05 µM, 0.1 µM, 0.25 µM, 0.5 µM, 1 µM, 1.25 µM, 2.5 µM, 5 µM, 10 µM, 20 µM and 50 µM. All drugs were dissolved in DMSO to prepare stock solutions. Cells were treated with 2 mL of desired drug solution per well in duplicate and incubated at 37 °C with 5% CO_2_ for 96 h. Drug solutions were removed, and wells were washed twice with 1 × phosphate-buffered saline (PBS; Lonza). Fresh complete media were added to the plates, and cells were allowed to grow for an additional 10 days at 37 °C with 5% CO_2_. Clones were washed twice and fixed with 4% paraformaldehyde/formaldehyde for 10 min at room temperature (RT). Clones were stained with 0.5% crystal violet solution (Pro-Lab diagnostics PL700; Richmond Hill, ON, Canada) for 10 min–2 h at RT, shaken, washed and dried (once desired staining was achieved). Plates were imaged using the GelCount™ system (Oxford Optronix; Oxford, UK) and analyzed using the ColonyCountJ Plugin (kindly shared by Dr. Dharmendra Kumar Maurya, Mumbai, India) in ImageJ v1.53e [100]. Statistical analysis was performed using one-way ANOVA with Dunnett’s Test for Multiple Comparisons in GraphPad Prism v7.00 for Windows (GraphPad Software, San Diego, CA, USA). A *p* value of < 0.05 was considered statistically significant. Experiments were performed in triplicates/quadruplicates.

### 4.3. MTT Assay

Effect of ACY-1215 on OMM2.5 cell viability was determined using 3-(4,5-dimethylthiazol-2-yl)-2,5-diphenyltetrazolium bromide) MTT assay, as described previously [38]. Briefly, 5000 cells/well were seeded into 96-well plates and allowed to adhere overnight. Cells were treated in triplicates with either 0.5% DMSO (vehicle control) or 15% H_2_O_2_ or 0.5, 1, 5, 10, 20 and 50 µM ACY-1215 (SelleckChem), prepared in complete media for 96 h. Drug solution was removed, MTT (3-(4,5-dimethylthiazol-2-yl)-2,5-diphenyltetrazolium bromide) dye and serum-free media were added in a 1:10 ratio to each well and incubated in the dark for 2.5 h at 37 °C. Following this, 100% DMSO (1:1 ratio) was added to each well, and absorbance was measured at 570 nm using a SpectraMax^®^ M2 microplate reader (Molecular Devices Corporation, Sunnyvale, CA, USA). One-way ANOVA with Dunnett’s Test for Multiple Comparisons statistical analysis IC_50_ value for ACY-1215 was calculated using GraphPad Prism v8.00 (GraphPad Software, San Diego, CA, USA).

### 4.4. OMM2.5 Zebrafish Xenografts

Zebrafish rearing and husbandry were performed in accordance with ethical regulations of the Linköping Animal Research Ethics Committee. Only larval, and not animal forms, of zebrafish were used in the study Zebrafish embryos/larvae from *Tg(fli1a:EGFP)* background were raised in embryo media containing 5 mM NaCl, 0.17 mM KCl, 0.33 mM MgCl_2_, 0.33 mM CaCl_2_ and 0.003% phenylthiourea (PTU), in a Petri dish at 28.5 °C incubator. Adult *Tg(fli1a:EGFP)* zebrafish were maintained in a 14 h light/10 h dark cycle in a recirculating water system at 28 °C. OMM2.5 cells were prepared for transplantation, as described in a previously published report [101]. OMM2.5 cells were labeled with 6 mg/mL Dil (Sigma-Aldrich) stain solution prepared in 1× PBS for 30 min at 37 °C. OMM2.5 Dil-labeled cells were washed twice with 1× PBS and resuspended in complete media. OMM2.5 Dil-labeled cells were filtered through a 40 µm cell strainer prior to microinjection. Approximately 200–500 labeled cells were microinjected (microINJECTOR^TM^, Tritech Research; Los Angeles, CA, USA) into the perivitelline space of 2-day-old *Tg(fli1a:EGFP)* zebrafish larvae under anesthesia (0.05 mg/mL MS222; Sigma-Aldrich). Larvae were imaged using a fluorescent microscope (SMZ1500 attached to DS-Fi2 camera head, Nikon; Tokyo, Japan) for red fluorescence and placed individually into 48-well plates. Only larvae with tumor cells correctly implanted in the perivitelline space were included in the study. Larvae (0 days post treatment (dpt) were treated with either 0.5% DMSO, 20 µM ACY-1215 (MCE, MedChemExpress; NJ, USA), 20 µM Dacarbazine (TCI, Tokyo Chemical Industry; Tokyo, Japan) or 1.25 µM ML329 for 3 days at 35 °C and imaged at both perivitelline space and caudal vein plexus post treatment (25–32 pooled larvae were used per treatment group). Differences in transplanted OMM2.5 Dil-labeled cells’ primary fluorescence between 0 dpt and 3 dpt were measured, normalized and calculated using ImageJ. Before drug treatment, toxicity assays were performed with either 0.5% DMSO, ACY-1215, Dacarbazine or ML329 (ranges from 1–100 µM). A total of 8 larvae (4 larvae/well) per treatment group were exposed to the desired concentration of drug solutions for 3 days in 24/48-well plates at 35 °C and imaged at 3 dpt. One-way ANOVA with Dunnett’s Test for Multiple Comparisons or Student’s T test statistical analyses were performed using GraphPad Prism v7.00 (GraphPad Software, San Diego, CA, USA).

### 4.5. Proteome Profiling and Mass Spectrometry Analysis

OMM2.5 cells were seeded at a density of 1 × 10^6^ cells per well and drug treated for 4 or 24 h with 0.5% DMSO or 20 µM ACY-1215, in duplicate (*N* = 4). Protein was isolated using PreOmics iST for protein/proteomics preparation kit (PreOmics GmbH; Martinsried, Germany), according to manufacturer’s protocol. Mass spectrometry and bioinformatic analysis of samples were performed as described previously [102]. Slight variations to methodology consisted of raw data processing performed with MaxQuant v1.6.10.43, with MS/MS spectra and database search performed against Uniprot *Homo sapiens* database (2020_05) containing 75,074 entries [102]. Pathway analysis of enriched proteins (a fold change of (+/−) ≥ 1.2 and a *p* value of ≤ 0.05) was performed using ClueGo (v2.5.8) [103] and Cluepedia (v1.5.8) [104] plugins in Cytoscape (v3.8.2) [105], with the *Homo sapiens* (9606) marker set. GO: Biological Process functional pathway databases, consisting of 18,058 genes, were used. GO tree levels (min = 3; max = 8) and GO term restriction (min#genes = 3, min% = 4%) were set, and terms were grouped using a Kappa Score Threshold of 0.4. The classification was performed by two-sided hypergeometric enrichment test, and its probability value was corrected by the Benjamini–Hochberg method (Adjusted % Term *p*-value < 0.05).

### 4.6. Western Blot Analysis

Protein was isolated from Mel270, Mel285, OMM2.5 and ARPE19 cells at a cell density of 1 × 10^6^ or 4 × 10^5^, and immunoblotting was performed as described [38]. For validation of proteomics data, protein isolated for MS study was utilized. Protein concentrations were measured using BCA protein assay kit (ThermoFisher Scientific; Waltham, MA, United States) in accordance with manufacturer’s instructions, and 10 μg of protein was loaded per lane (N = 3–4). Blots were probed for HDAC6 (1:1000; #7558, Cell Signaling Technology; Danvers, MA, USA, kindly provided by Dr. Tríona Ní Chonghaile, Dublin, Ireland), acetylated α-tubulin (1:1000; #5335, Cell Signaling Technology), MITF (1:1000; #ab122982, Abcam kindly provided by Dr. Desmond Tobin, Dublin, Ireland), cleaved PARP (1:1000; #5625, Cell Signaling Technology kindly provided by Dr. Emma Dorris, Dublin, Ireland), p-ERK (1:500; #sc-7383, Santa Cruz Biotechnology Inc.; Dallas, TX, USA), ERK (1:500; #sc-514302, Santa Cruz Biotechnology, Inc.), α-tubulin (1:1000; #sc-5286, Santa Cruz Biotechnology Inc.), GAPDH (1:1000; #2118, Cell Signaling Technology) and β-actin (1:1000; #A5441, Sigma-Aldrich). Anti-rabbit IgG, HRP-linked Antibody (1:3000; #7074s, Cell Signaling Technology) and anti-mouse IgG, HRP-linked Antibody (1:3000; #7076s, Cell Signaling Technology) were used as secondary antibodies. Signal was detected with enhanced chemiluminescence substrate (Pierce™ ECL Western Blotting Substrate; ThermoFisher Scientific). Densitometry analysis was performed using ImageJ and one-way ANOVA with Dunnett’s Test for Multiple Comparisons or Student’s T test statistical analysis using GraphPad Prism v7.00 (GraphPad Software, San Diego, CA, USA).

### 4.7. Flow Cytometry Analysis

A total of 300,000 OMM2.5 cells were seeded and treated with 0.5% DMSO, 50 µM Etoposide (Sigma-Aldrich; kindly provided by Dr. William Watson, Dublin, Ireland), 10, 20 and 50 µM ACY-1215 or 20 µM Dacarbazine, in duplicate (N = 3–4) for 4, 24 and 96 h at 37 °C with 5% CO_2_. Cells were trypsinized and filtered through 50 µm CellTrics filter. Live cells were labeled sequentially with YO-PRO™-1 Iodide (Molecular Probes^TM^ by ThermoFisher Scientific; Waltham, MA, United States) for 15 min and Propidium Iodide (PI, Molecular Probes^TM^ by ThermoFisher Scientific) for 3 min, in the dark, at RT, to analyze apoptotic events. For cell cycle analysis, cells were fixed in ice-cold 70% ethanol at 4 °C. After being fixed, cells were labeled with 1.25 µL of 1 mg/mL PI stock and co-treated with 2.5 µL of 10 mg/mL RNase A enzyme (ThermoFisher Scientific) for 30 min at RT, in the dark. All samples were run on a BD Accuri^TM^ C6 Flow Cytometer (BD Biosciences; NJ, USA), and up to 50,000 events were recorded per sample (N = 3–4). YO-PRO™-1 Iodide and PI were excited using a 488 nm laser, and its fluorescence was collected using FL-1 channel (B530/30 band pass filter) and FL-3 channel (B675LP band pass filter), respectively. For cell cycle analysis, PI was excited using a 488 nm laser and its fluorescence collected using FL-2 channel (575/25 band pass filter). The collected samples were gated based on controls (DMSO/Etoposide) and preliminarily analyzed using CFlow Plus Software (v1.0.264.21; BD Biosciences; NJ, USA). Reanalysis was performed using FCS Express^TM^ De Novo (Research Edition) v6 software. The instrument was calibrated with manufacturer’s specifications prior to use. Two-way ANOVA followed by Tukey’s Multiple Comparison test or Dunnett’s Test for Multiple Comparisons statistical analyses were performed using GraphPad Prism v7.00 (GraphPad Software, San Diego, CA, USA). Detailed information on flow cytometry experiments and analysis performed are provided in Table S3.

### 4.8. TCGA Analysis

Survival analyses were performed with package “survminer”, R v3.5.0 (R Foundation for Statistical Computing, Vienna, Austria). Gene expression and clinical data from 80 primary UM included in The Cancer Genome Atlas (TCGA) were collected from the cBioPortal. RNA-seq data were downloaded in Fragments Per Kilobase of exon per million fragments Mapped (FPKM) and then converted to log2 scale. The associations between gene expression and prognosis were assessed by Cox proportional hazard regression models. Progression-Free Survival (PFS) and Overall Survival (OS) were used as end points. For categorization of the gene expression into “High” and “Low” categories, median values were used as cut-off. Survival probabilities were plotted on a Kaplan–Meier curve, and a Log-rank test was used to compare the two groups. Progression-free survival is defined as time until metastatic recurrence. Overall survival is defined as death by any cause.

## 5. Conclusions

This research provides evidence that ACY-1215 and ML329 should be further investigated to establish their potential as treatment option(s) for MUM. Specifically, this study proves the efficacy of ACY-1215 as an anti-cancer agent for MUM cells, OMM2.5, in vitro and in vivo. We have additionally elucidated that ACY-1215 treatment reduces MITF expression in OMM2.5 cells.

## Data Availability

Access to raw datasets will be provided upon request to the corresponding author.

## Supplementary Materials

The following supporting information can be downloaded at: https://www.mdpi.com/article/10.3390/cancers14030782/s1, Figure S1: HDAC6 inhibitors present with anti-cancer activity in UM (Mel285, Mel270) and MUM (OMM2.5) cell lines. Figure S2: Toxicity effects of ACY-1215 and Dacarbazine in zebrafish larvae. Figure S3: Raw Western blot images for HDAC6 expression in UM and MUM cells. Figure S4: Potential off-target effects of ACY-1215 on HDAC isozymes. Figure S5: Proteome profile of OMM2.5 cells treated with ACY-1215 for 4 h. Figure S6: Pathway analysis map of down and upregulated proteins following ACY-1215 treatment for 24 h. Figure S7: Raw Western blot images for acetylated α-tubulin, MITF, p-ERK, ERK and cleaved PARP expression in ACY-1215-treated OMM2.5 cells. Figure S8: DNA ploidy in OMM2.5 cells. Figure S9: 4 h ACY-1215 treatment did not have a profound effect on apoptosis pathway in OMM2.5 cells. Figure S10: Toxicity screen of ML329 in zebrafish larvae. Table S1: List of downregulated proteins and associated pathways after 24 h of ACY-1215 treatment. Table S2: List of upregulated proteins and associated pathways following ACY-1215 treatment for 24 h. Table S3: Minimum Information about a Flow Cytometry Experiment (MIFlowCyt).
