# Supplementary material for: Uveal Melanoma Cell Line Proliferation Is Inhibited by Ricolinostat, a Histone Deacetylase Inhibitor
Source: Cancers (Basel). 2022 Feb 3;14(3):782. doi: 10.3390/cancers14030782 (PMC8833954; doi:10.3390/cancers14030782)
Supplement: Supplementary file 1 [file cancers-14-00782-s001.zip › cancers-1536201-SI.pdf]

Uveal Melanoma Cell Line Proliferation Is Inhibited by Ricolinostat, a Histone Deacetylase Inhibitor

Supplementary Figures and Tables

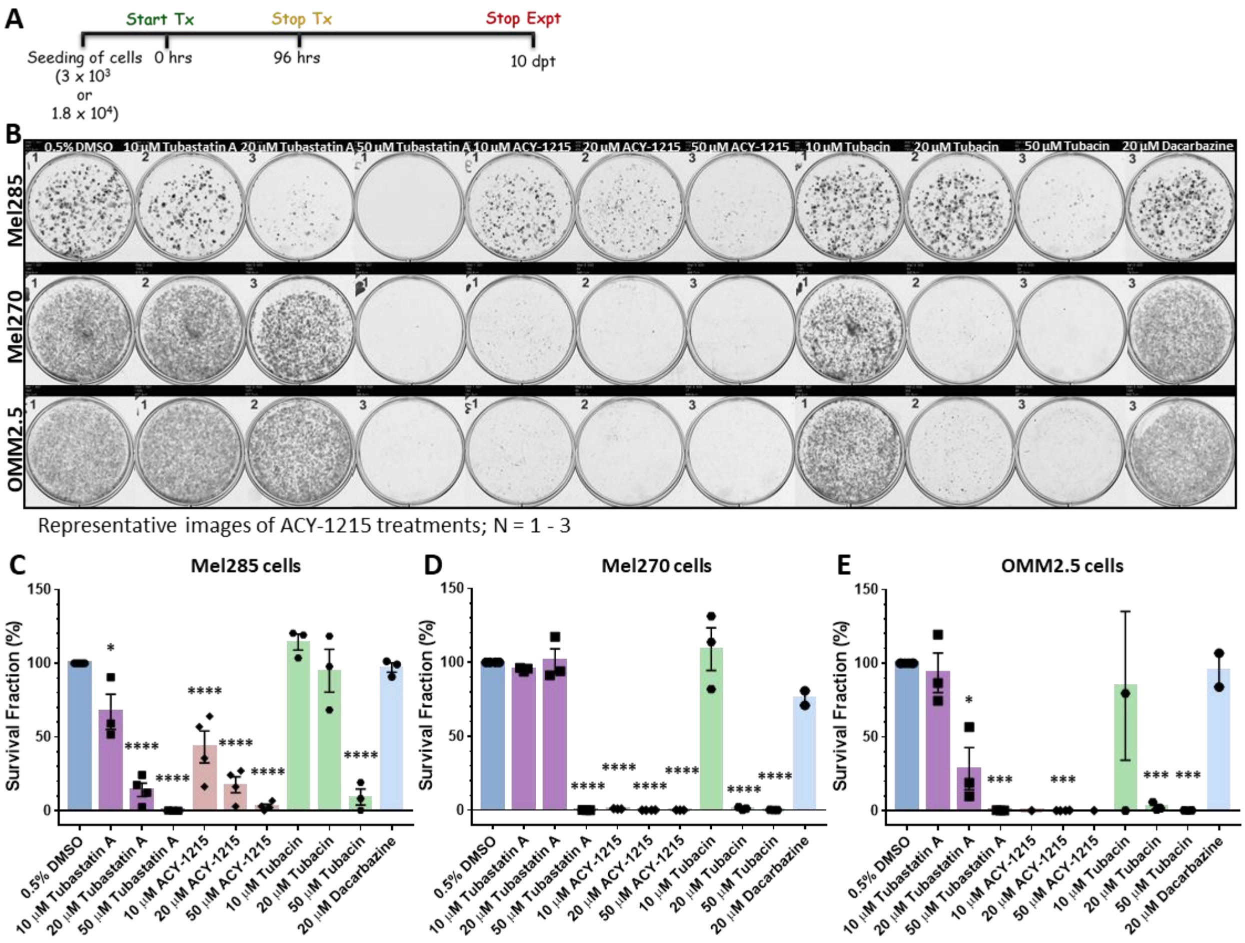

Figure S1: HDAC6 inhibitors present with anti-cancer activity in UM (Mel285, Mel270) and MUM (OMM2.5) cell lines. A: Schematic diagram illustrating treatment regime used. B: Representative image of clonogenic assay plates for Mel285 (top panel), Mel270 (middle panel) and OMM2.5 cells (bottom panel) treated with 0.5% DMSO, 10, 20 or 50 μM Tubastatin A, 10, 20 or 50 μM ACY-1215, 10, 20 or 50 μM Tubacin or 20 μM Dacarbazine for 96 hours. C, D and E: A dose-dependent decrease in surviving fraction of colonies was observed across the three different HDAC6i tested in comparison to 0.5% DMSO treatment. Percentage of surviving colonies was comparable between 20 μM Dacarbazine and 0.5% DMSO treatment group. One-way ANOVA with Dunnett's Test for Multiple Comparisons statistical analysis was performed, error bars represent mean ± SEM, ****p = 0.0001 (N = 2 - 4).

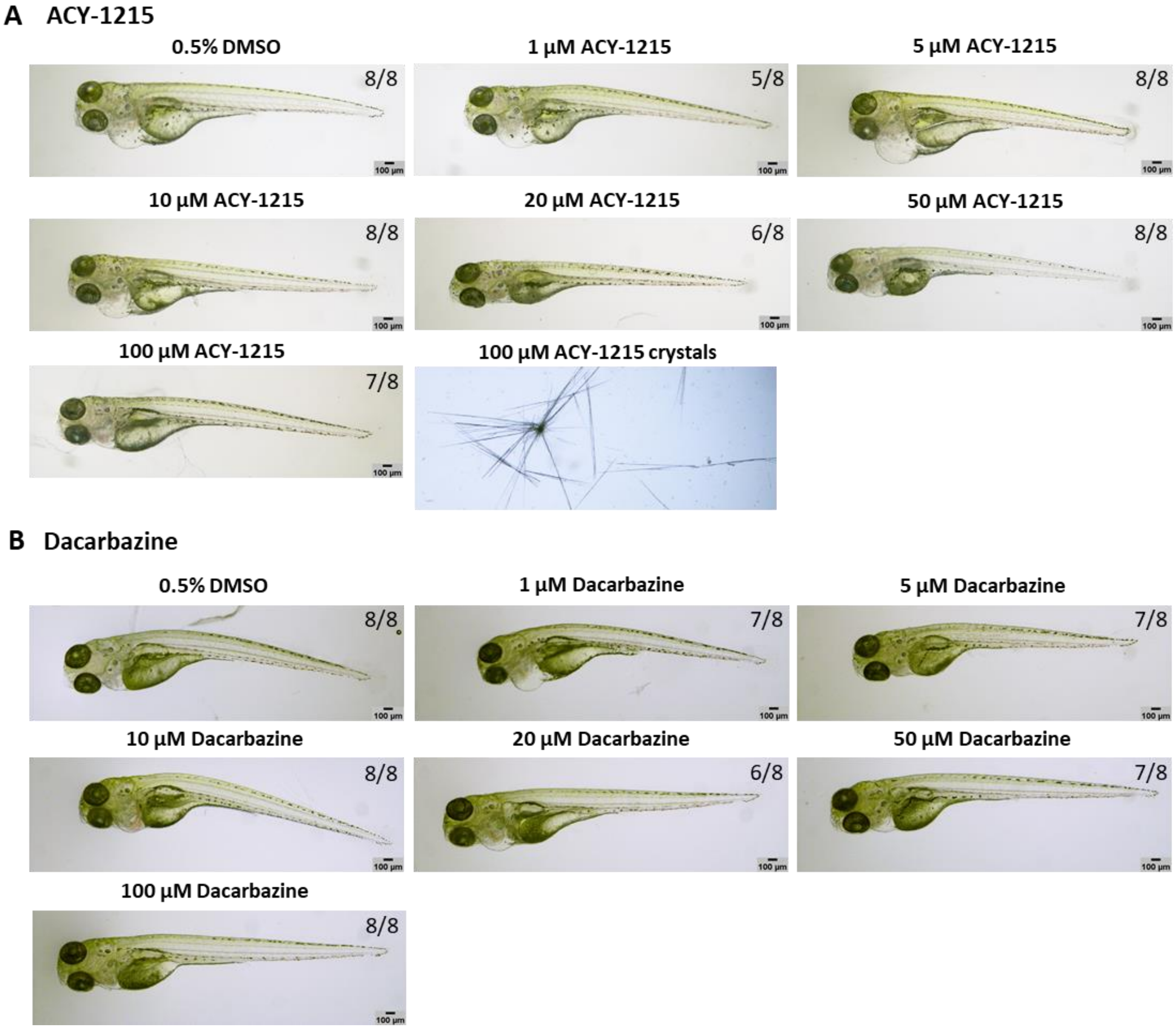

Figure S2: Toxicity effects of ACY-1215 and Dacarbazine in zebrafish larvae. A and B: Increasing doses of ACY-1215 and Dacarbazine, up to 100 µM, are well tolerated by 5 days (3 days post treated) old *Tg(fli1a:EGFP)* zebrafish larvae (n = 8 per treatment group). Drug crystals were observed to form at 100 µM ACY-1215.

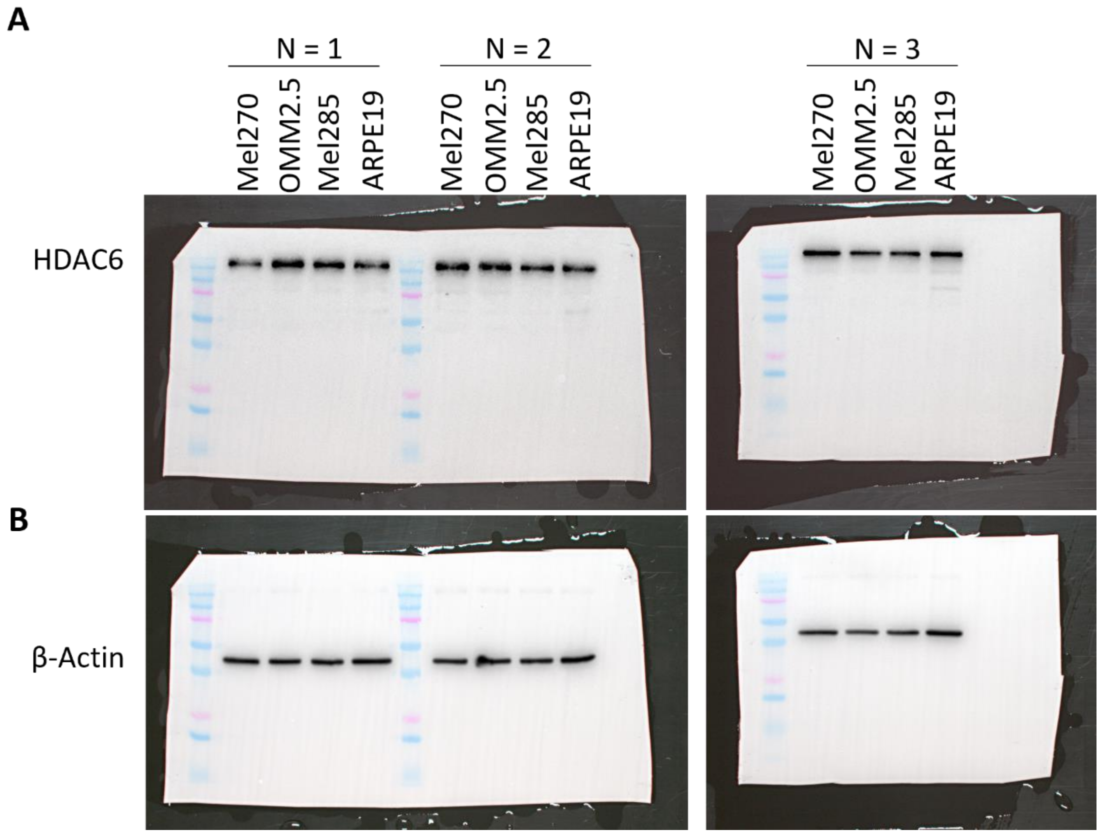

Figure S3: Raw Western blot images for HDAC6 expression in UM and MUM cells. A: HDAC6 expression in UM (Mel270, Mel285) and mUM (OMM2.5) cells, is comparable to ARPE19 cells (N = 3). B: β-actin was used as loading control.

A

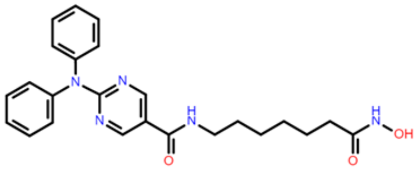

ACY-1215 (Ricolinostat)

B

HDAC	IC_{50}	Activity	HDAC	IC_{50}	Activity
HDAC6	4.7 nM	Potent & Selective	HDAC5	5 µM	Minimal
HDAC2	48 nM	Less Active	HDAC4	7 µM	Minimal
HDAC3	51 nM	Less Active	HDAC9	> 1 µM	Minimal
HDAC1	58 nM	Less Active	HDAC11	> 1 µM	Minimal
HDAC8	100 nM	Slight	Sirt 1	> 1 µM	Minimal
HDAC7	1.4 µM	Minimal	Sirt 2	> 1 µM	Minimal

Adapted from SelleckChem

C

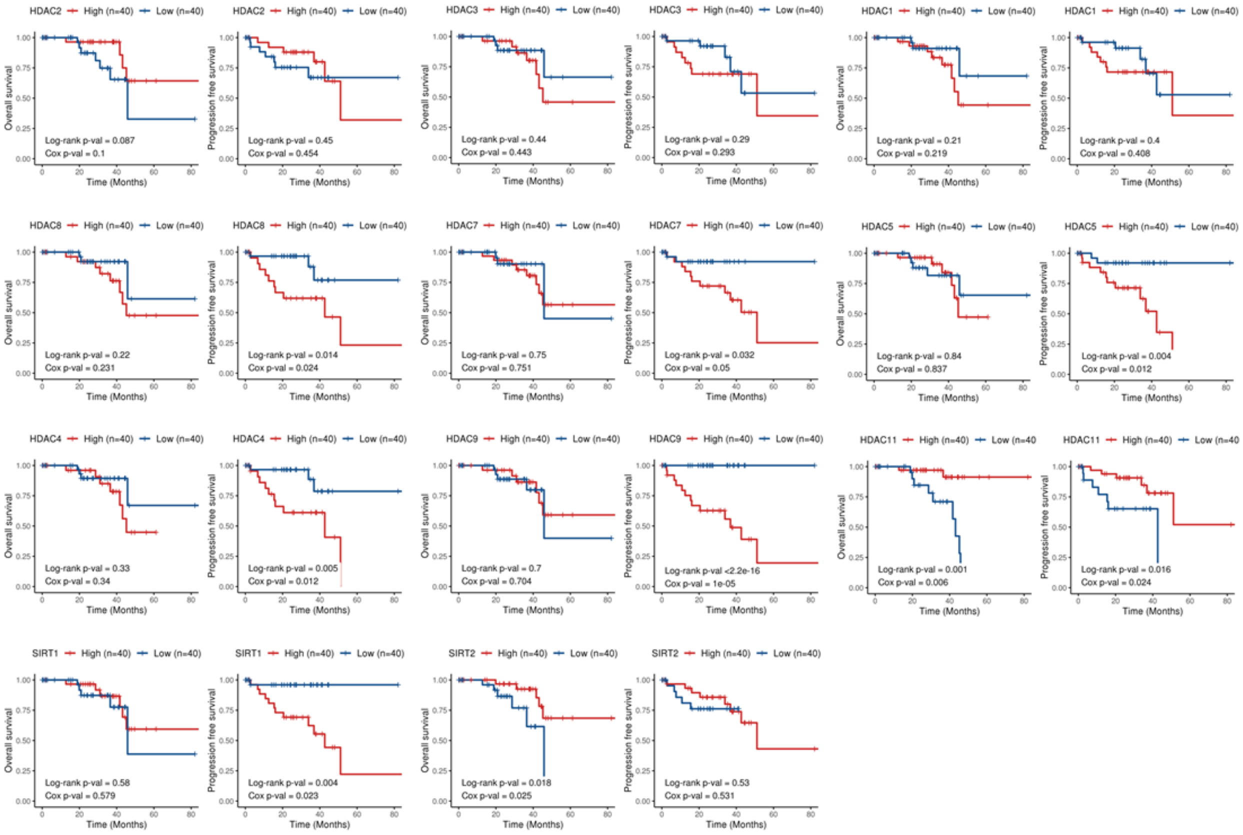

Figure S4: Potential off-target effects of ACY-1215 on HDAC isozymes. A: Chemical structure of ACY-1215 drawn using ChemSpider. B: Table highlighting ACY-1215 IC_{50} values against various HDAC isozymes. C: Kaplan-Meier survival curves demonstrating correlation between expression of various HDAC isoforms and overall survival (OS) or progression free survival (PFS) in UM patients. Median values were used as cut-off for high (red) and low (blue) expression levels, with Log-rank *p*-values (categorical variable) and Cox *p*-values (continuous variable) calculated (n = 80).

A

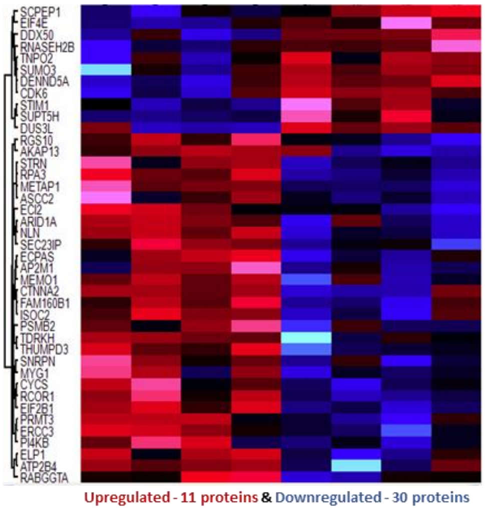

B

Top 10 Downregulated Proteins

Gene	UNIPROT	Protein Names	-Log Student's T-test p-value	Log Student's T-test Difference	Fold Change
CTNNA2	P26232	Catenin alpha-2	1.78	− 3.80	− 13.96
AKAP13	Q12802	A-kinase anchor protein 13	1.44	− 1.90	− 3.74
ARID1A	O14497	AT-rich interactive domain-containing protein 1A	1.71	− 1.86	− 3.63
NLN	Q9BYT8	Neurolysin, mitochondrial	2.87	− 1.54	− 2.92
RCOR1	Q9UKL0	REST corepressor 1	2.31	− 1.47	− 2.77
ELP1	F5H2T0	IkappaB kinase complex-associated protein	1.65	− 1.37	− 2.59
SNRPN	J3KRY3	Small nuclear ribonucleoprotein-associated protein N (Fragment)	1.46	− 0.82	− 1.76
PRMT3	O60678	Protein arginine N-methyltransferase 3	1.63	− 0.77	− 1.71
ATP2B4	P23634	Plasma membrane calcium-transporting ATPase 4	1.39	− 0.69	− 1.62
TDRKH	Q5SZR4	Tudor and KH domain containing, isoform CRA_a	1.37	− 0.62	− 1.54

Top 10 Upregulated Proteins

Gene	UNIPROT	Protein Names	-Log Student's T-test p-value	Log Student's T-test Difference	Fold Change
DDX50	A0A087WVC1	RNA helicase	2.02	1.40	2.64
DUS3L	Q96G46	tRNA-dihydrouridine(47) synthase [NAD(P)(+)]-like	1.62	1.16	2.24
SUMO3	P55854	Small ubiquitin-related modifier 3	1.40	1.14	2.20
DENND5A	H0YDE6	DENN domain-containing protein 5A (Fragment)	2.03	0.44	1.36
SCPEP1	Q9HB40	Retinoid-inducible serine carboxypeptidase	1.76	0.36	1.28
TNPO2	A0A075B780	Transportin-2	1.35	0.32	1.25
SUPT5H	O00267	Transcription elongation factor SPT5	1.58	0.29	1.22
RNASEH2B	A0A2R8YCX2	Ribonuclease H2 subunit B	1.41	0.22	1.17
EIF4E	P06730	Eukaryotic translation initiation factor 4E	1.51	0.12	1.09
CDK6	Q00534	Cyclin-dependent kinase 6	1.74	0.12	1.09

C Downregulated proteins - Biological Processes

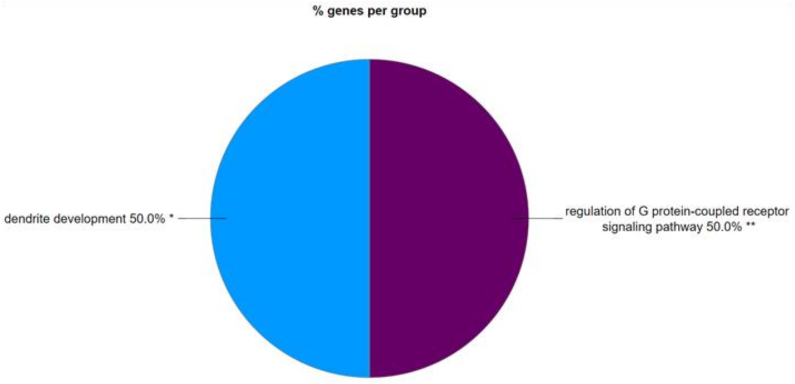

Figure S5: Proteome profile of OMM2.5 cells treated with ACY-1215 for 4 hours. A: Heat map presenting significantly differentially expressed proteins after 4 hours of 20 µM ACY-1215 treatment in OMM2.5 cells. 30 (blue) proteins were downregulated, and 11 (red) proteins were upregulated (N = 4). B: Table showing the top 10 down- and up-regulated proteins at 4 hours. C: Enriched protein pathway analysis for GO term: biological processes, identified dendrite development and regulation of G protein-coupled receptor signaling pathway to be significantly downregulated.

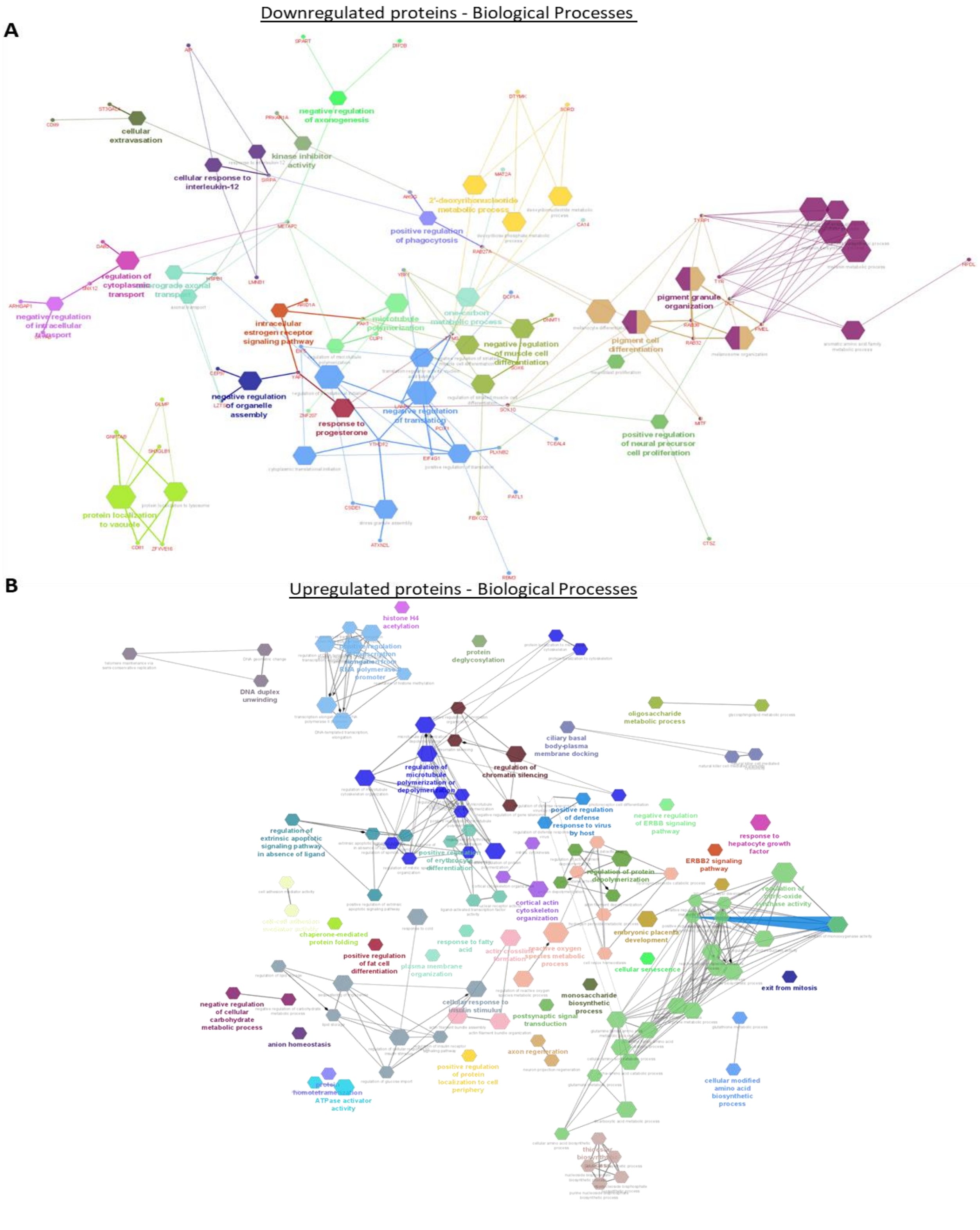

Figure S6: GO: Biological processes pathway analysis map of down- and up-regulated proteins following ACY-1215 treatment for 24 hours.

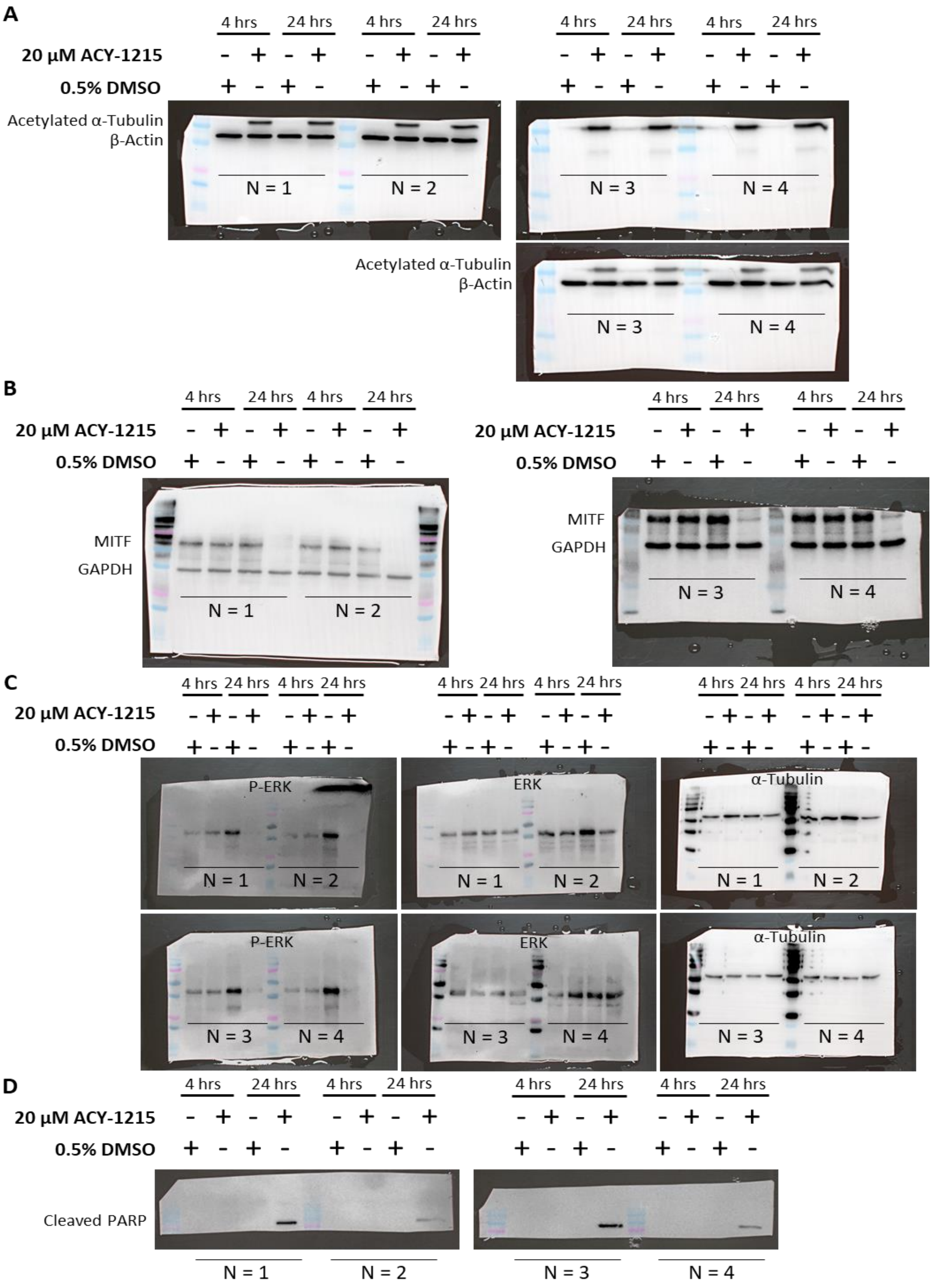
A
4 hrs
24 hrs
20 µM ACY-1215
0.5% DMSO
Acetylated α-Tubulin
β-Actin
N = 1
N = 2
N = 3
N = 4
B
MITF
GAPDH
C
P-ERK
ERK
α-Tubulin
D
Cleaved PARP

Figure S7: Raw Western blot images for acetylated α-tubulin, MITF, p-ERK, ERK and cleaved PARP expression in ACY-1215 treated OMM2.5 cells. A: Acetylated α-tubulin expression is upregulated after 4 and 24 hours of 20 µM ACY-1215 treatment in OMM2.5 cells. B: MITF expression remained unchanged after 4 hours of 20 µM ACY-1215 treatment. 24 hours of 20 µM ACY-1215 resulted in a significant reduction in MITF expression levels compared to vehicle control. C: p-ERK/Total ERK expression levels not altered following 4 hours of treatment with 20 µM ACY-1215. 20 µM ACY-1215 treatment for 24 hours led to a significant reduction in p-ERK expression levels, in comparison to vehicle control. D: Cleaved PARP expression was upregulated in OMM2.5 cells 24 hours post treated with 20 µM ACY-1215. β-actin, α-tubulin and GAPDH were used as loading control (N = 4).

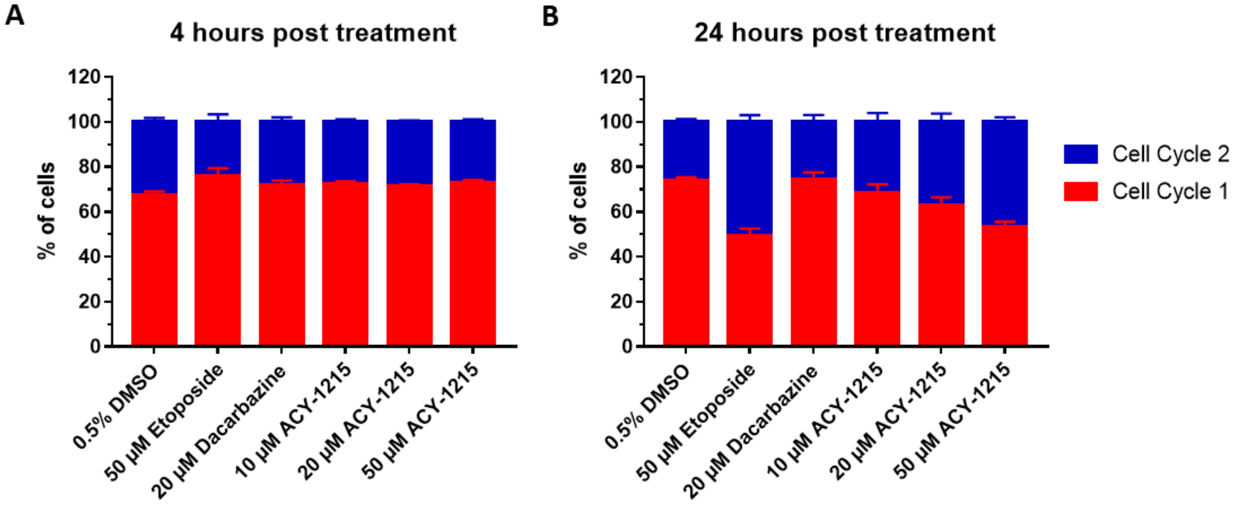

Figure S8: DNA ploidy in OMM2.5 cells. A and B: OMM2.5 cells display DNA ploidy as presented by the two cell cycle populations observed (N = 4). Error bars indicate mean + SEM (N = 3)

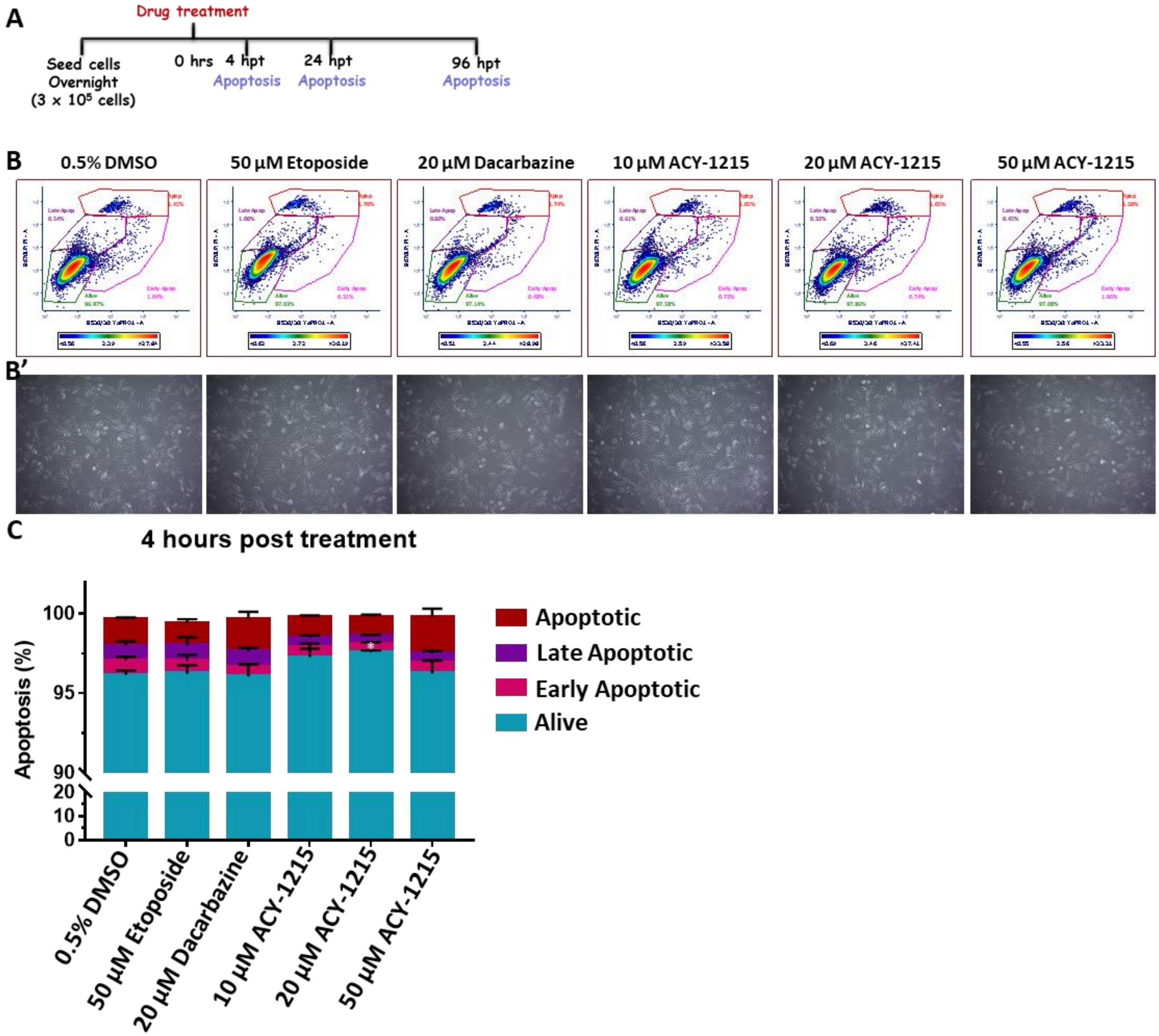

Figure S9: 4 hours ACY-1215 treatment did not have a profound effect on apoptosis pathway in OMM2.5 cells. A: Illustration of treatment regime. B: Representative plots of treated OMM2.5 cell singlets gating in different stages of apoptosis. B': Micrographs corresponding to OMM2.5 drug treatment groups at 4 hours. C: No change was observed in live, early, late and apoptotic cell populations following treatment with Etoposide and all concentrations of ACY-1215 treatment. On average, a significant increase in percentage of live cells was detected following 20 µM ACY-1215 (*, $p = 0.03$) treatment compared to 0.5% DMSO treatment. Two-way ANOVA followed by Tukey's Multiple Comparisons statistical test was performed, error bars show mean + SEM (N = 3).

Supplementary Fig 10: Toxicity screen in zebrafish

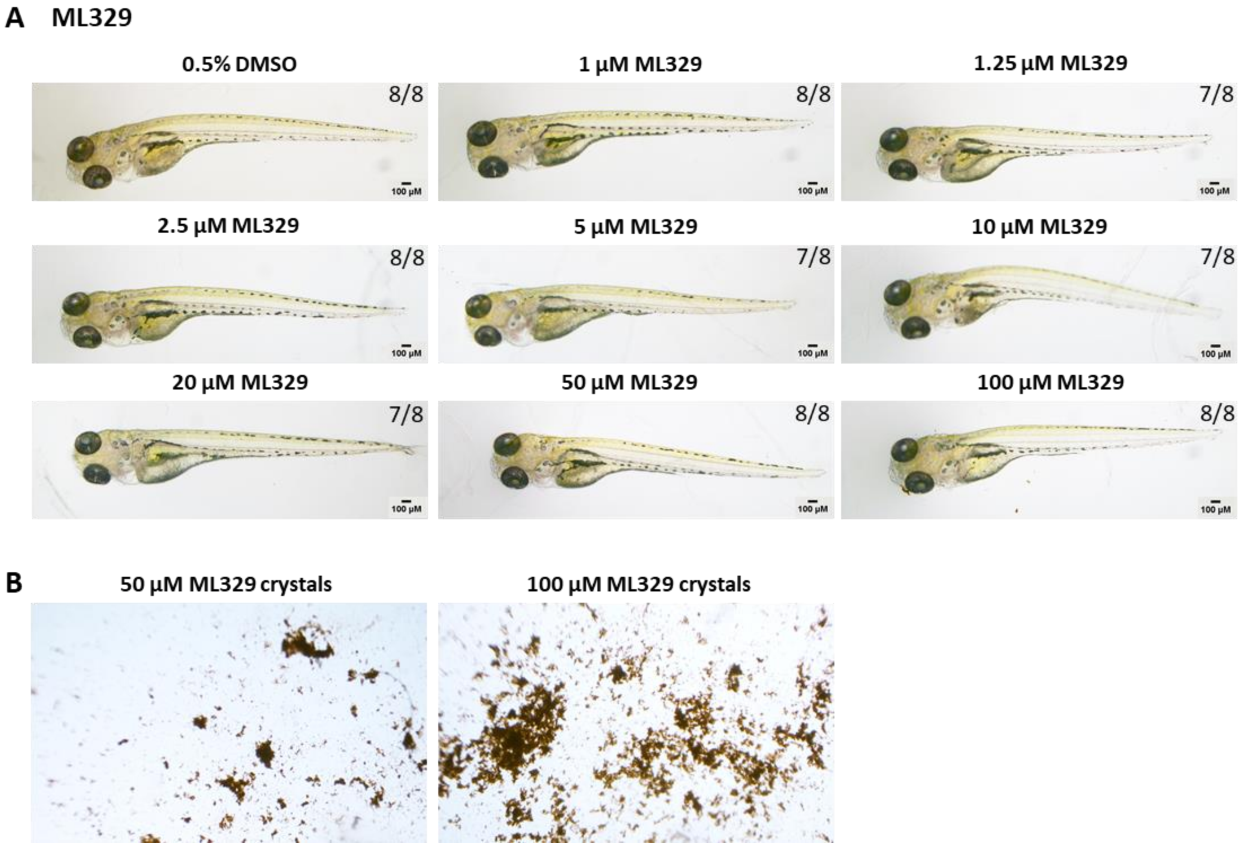

Figure S10: Toxicity screen of ML329 in zebrafish larvae. A: Increasing doses of ML329, up to 100 µM, was well tolerated by 5 days (3 days post treated) old *Tg(fli1a:EGFP)* zebrafish larvae (n = 8 per treatment group). B: Drug precipitates observed in embryo media at 50 and 100 µM ML329 concentrations 3 days post treatment.

Table S1: List of downregulated proteins and associated pathways after 24 hours of ACY-1215 treatment.

ID	Term	Associated Genes Found
GO:0019210	Kinase inhibitor activity	AHSG, HSPB1, PRKAR1A
GO:0050771	Negative regulation of axonogenesis	DIP2B, METAP2, SPART
GO:0006730	One-carbon metabolic process	CA14, MAT2A, TYMS
GO:1902116	Negative regulation of organelle assembly	CEP97, LZTS1, YAP1
GO:1903649	Regulation of cytoplasmic transport	DAB2, METAP2, SNX12
GO:0030520	Intracellular estrogen receptor signaling pathway	ARID1A, PAK1, YAP1
GO:0032387	Negative regulation of intracellular transport	ARHGAP1, CRYAB, SNX12
GO:0032570	Response to progesterone	SOX10, TYMS, YAP1
GO:0045123	Cellular extravasation	CD99, SIRPA, ST3GAL4
GO:0050766	Positive regulation of phagocytosis	AHSG, RAB27A, SIRPA
GO:0070671	Response to interleukin-12	AIP, LMNB1, SIRPA
GO:0071349	Cellular response to interleukin-12	AIP, LMNB1, SIRPA
GO:0072665	Protein localization to vacuole	CD81, GLMP, GNPTAB, SH3GLB1, ZFYVE16
GO:0061462	Protein localization to lysosome	CD81, GLMP, GNPTAB, ZFYVE16
GO:0098930	Axonal transport	HSPB1, LZTS1, METAP2
GO:0008089	Anterograde axonal transport	HSPB1, LZTS1, METAP2
GO:2000179	Positive regulation of neural precursor cell proliferation	CTSZ, DCT, SOX10
GO:0007405	Neuroblast proliferation	DCT, PLXNB2, SOX10
GO:0046785	Microtubule polymerization	CLIP1, METAP2, PAK1, ZNF207
GO:0031113	Regulation of microtubule polymerization	CLIP1, METAP2, PAK1
GO:0019692	Deoxyribose phosphate metabolic process	DTYMK, SORD, TYMS
GO:0009262	Deoxyribonucleotide metabolic process	DTYMK, SORD, TYMS
GO:0009394	2'-deoxyribonucleotide metabolic process	DTYMK, SORD, TYMS
GO:0051148	Negative regulation of muscle cell differentiation	DNMT1, PAK1, SOX6, YBX1
GO:0051153	Regulation of striated muscle cell differentiation	FBXO22, PAK1, SOX6, YBX1
GO:0051154	Negative regulation of striated muscle cell differentiation	PAK1, SOX6, YBX1
GO:0050931	Pigment cell differentiation	MITF, RAB27A, RAB32, RAB38, SOX10, TYRP1
GO:0030318	Melanocyte differentiation	MITF, RAB27A, SOX10, TYRP1

ID	Term	Associated Genes Found
GO:0048753	Pigment granule organization	PMEL, RAB32, RAB38, TYRP1
GO:0032438	Melanosome organization	PMEL, RAB32, RAB38, TYRP1
GO:0034063	Stress granule assembly	ATXN2L, CSDE1, YTHDF2
GO:0006446	Regulation of translational initiation	CSDE1, EIF4G1, EIF5, HSPB1, LARP1, THDF2
GO:0002183	Cytoplasmic translational initiation	EIF4G1, EIF5, YTHDF2
GO:0017148	Negative regulation of translation	DCP1A, EIF4G1, LARP1, PATL1, PCIF1, TYMS, YBX1, YTHDF2
GO:0045727	Positive regulation of translation	EIF4G1, LARP1, PCIF1, PLXNB2, RBM3, YTHDF2
GO:0090079	Translation regulator activity, nucleic acid binding	EIF4G1, EIF5, LARP1, TCEAL4, TYMS
GO:0044550	Secondary metabolite biosynthetic process	DCT, PMEL, TYR, TYRP1
GO:0046148	Pigment biosynthetic process	DCT, PMEL, TYR, TYRP1
GO:0050931	Pigment cell differentiation	MITF, RAB27A, RAB32, RAB38, SOX10, TYRP1
GO:0006582	Melanin metabolic process	DCT, PMEL, TYR, TYRP1
GO:0009072	Aromatic amino acid family metabolic process	DCT, HPDL, TYR
GO:0048753	Pigment granule organization	PMEL, RAB32, RAB38, TYRP1
GO:0042438	Melanin biosynthetic process	DCT, PMEL, TYR, TYRP1
GO:0046189	Phenol-containing compound biosynthetic process	DCT, PMEL, TYR, TYRP1
GO:0032438	Melanosome organization	PMEL, RAB32, RAB38, TYRP1

Table S2: List of upregulated proteins and associated pathways following ACY-1215 treatment for 24 hours.

ID	Term	Associated Genes Found
Go:0006517	Protein deglycosylation	MAN2B1, OS9, SEL1L
Go:0090398	Cellular senescence	ARG2, MAP2K1, PAWR
Go:0007009	Plasma membrane organization	AHNAK2, BAIAP2L1, NDRG1, PRDX6, RAB3A, SLC9A3R1
Go:0010458	Exit from mitosis	ANLN, EPS8, UBE2S
Go:0035728	Response to hepatocyte growth factor	CRK, GCLC, LGMN
Go:0038128	Erbb2 signaling pathway	CDC37, GRB2, HSP90AA1
Go:0043967	Histone h4 acetylation	HAT1, IWS1, RUVBL1
Go:0045600	Positive regulation of fat cell differentiation	AAMDC, LCP1, STK4, SYAP1
Go:0046364	Monosaccharide biosynthetic process	ALDOC, G6PD, GOT1, SDHAF3, STK4
Go:0051289	Protein homotetramerization	ACOT13, GLS, TK1
Go:0055081	Anion homeostasis	ENPP1, GLS, SLC9A3R1
Go:0061077	Chaperone-mediated protein folding	DNAJB1, HSPA1A, SGTB
Go:0070542	Response to fatty acid	CAT, CCN2, LCP1
Go:0098926	Postsynaptic signal transduction	KPNA2, RGS10, STAT3
Go:1901185	Negative regulation of erbb signaling pathway	GRB2, LGMN, MVP
Go:1904377	Positive regulation of protein localization to cell periphery	ACSL3, EPB41L2, SORBS1
Go:0009311	Oligosaccharide metabolic process	GLA, MAN2B1, NEU1
Go:0006687	Glycosphingolipid metabolic process	ARSA, GLA, NEU1
Go:0031102	Neuron projection regeneration	CTNNA1, DHFR, MAP2K1
Go:0031103	Axon regeneration	CTNNA1, DHFR, MAP2K1
Go:0006749	Glutathione metabolic process	G6PD, GCLC, GGCT
Go:0042398	Cellular modified amino acid biosynthetic process	DHFR, GCLC, GGCT
Go:0045912	Negative regulation of carbohydrate metabolic process	ENPP1, STAT3, STK4
Go:0010677	Negative regulation of cellular carbohydrate metabolic process	ENPP1, STAT3, STK4
Go:0060590	Atpase regulator activity	ATP1B1, DNAJB1, RAB3A
Go:0001671	Atpase activator activity	ATP1B1, DNAJB1, RAB3A
Go:0001892	Embryonic placenta development	CCN1, GRB2, MAP2K1, MIA3, STK4
Go:0060711	Labyrinthine layer development	CCN1, GRB2, MAP2K1

ID	Term	Associated Genes Found
Go:0098631	Cell adhesion mediator activity	BAIAP2L1, CXADR, PDLIM5
Go:0098632	Cell-cell adhesion mediator activity	BAIAP2L1, CXADR, PDLIM5
Go:0000281	Mitotic cytokinesis	ANLN, ARL3, RACGAP1
Go:0030866	Cortical actin cytoskeleton organization	ANLN, EPB41L2, LCP1, RACGAP1
Go:0030865	Cortical cytoskeleton organization	ANLN, EPB41L2, LCP1, RACGAP1
Go:0032201	Telomere maintenance via semi-conservative replication	BLM, RFC1, RFC4
Go:0032392	Dna geometric change	BLM, CETN2, MNAT1, RFC4, RUVBL1
Go:0032508	Dna duplex unwinding	BLM, CETN2, MNAT1, RFC4, RUVBL1
Go:0002228	Natural killer cell mediated immunity	CRK, PRDX1, TUBB2A, TUBB4B
Go:0042267	Natural killer cell mediated cytotoxicity	CRK, PRDX1, TUBB2A, TUBB4B
Go:0097711	Ciliary basal body-plasma membrane docking	CEP131, CETN2, HSP90AA1, TUBB2A, TUBB4B
Go:0050688	Regulation of defense response to virus	HSP90AA1, SIN3A, TOMM70
Go:0050691	Regulation of defense response to virus by host	HSP90AA1, SIN3A, TOMM70
Go:0002230	Positive regulation of defense response to virus by host	HSP90AA1, SIN3A, TOMM70
Go:0051764	Actin crosslink formation	BAIAP2L1, EPS8, LCP1
Go:0061572	Actin filament bundle organization	BAIAP2L1, CCN2, EPS8, LCP1, LIMA1, PAWR, SORBS1
Go:0051017	Actin filament bundle assembly	BAIAP2L1, CCN2, EPS8, LCP1, LIMA1, PAWR, SORBS1
Go:0060969	Negative regulation of gene silencing	ATAD2, H1-10, STAT3
Go:1905268	Negative regulation of chromatin organization	ATAD2, H1-10, SIN3A, SUPT6H
Go:0006342	Chromatin silencing	ATAD2, H1-10, HAT1, SIN3A
Go:0031935	Regulation of chromatin silencing	ATAD2, H1-10, SIN3A
Go:0038034	Signal transduction in absence of ligand	CTNNA1, HSPA1A, PPP2R1B
Go:0097192	Extrinsic apoptotic signaling pathway in absence of ligand	CTNNA1, HSPA1A, PPP2R1B
Go:2001238	Positive regulation of extrinsic apoptotic signaling pathway	CTNNA1, PPP2R1B, STK4
Go:2001239	Regulation of extrinsic apoptotic signaling pathway in absence of ligand	CTNNA1, HSPA1A, PPP2R1B
Go:0098531	Ligand-activated transcription factor activity	MIA3, NR4A1, STAT3
Go:0045646	Regulation of erythrocyte differentiation	HSPA1A, MIA3, STAT3
Go:0045648	Positive regulation of erythrocyte differentiation	HSPA1A, MIA3, STAT3
Go:0004879	Nuclear receptor activity	MIA3, NR4A1, STAT3

ID	Term	Associated Genes Found
Go:1901879	Regulation of protein depolymerization	CKAP2, DSTN, EPS8, LIMA1, METAP1
Go:0051261	Protein depolymerization	CKAP2, DSTN, EPS8, LIMA1, METAP1
Go:0030042	Actin filament depolymerization	DSTN, EPS8, LIMA1
Go:0030834	Regulation of actin filament depolymerization	DSTN, EPS8, LIMA1
Go:0035384	Thioester biosynthetic process	ACSL3, GCDH, PDP1
Go:0033866	Nucleoside bisphosphate biosynthetic process	ACSL3, GCDH, PDP1
Go:0034033	Purine nucleoside bisphosphate biosynthetic process	ACSL3, GCDH, PDP1
Go:0034030	Ribonucleoside bisphosphate biosynthetic process	ACSL3, GCDH, PDP1
Go:0071616	Acyl-coa biosynthetic process	ACSL3, GCDH, PDP1
Go:0032968	Positive regulation of transcription elongation from RNA polymerase ii promoter	BRD4, GTF2F1, SUPT6H
Go:0006354	DNA-templated transcription, elongation	BRD4, GTF2F1, HTATSF1, IWS1, MNAT1, SUPT6H
Go:0031060	Regulation of histone methylation	BRD4, IWS1, SUPT6H
Go:0032784	Regulation of DNA-templated transcription, elongation	BRD4, GTF2F1, HTATSF1, SUPT6H
Go:0006368	Transcription elongation from rna polymerase ii promoter	BRD4, GTF2F1, IWS1, MNAT1, SUPT6H
Go:0032786	Positive regulation of dna-templated transcription, elongation	BRD4, GTF2F1, SUPT6H
Go:0034243	Regulation of transcription elongation from rna polymerase ii promoter	BRD4, GTF2F1, SUPT6H
Go:0072593	Reactive oxygen species metabolic process	ARG2, CAT, CBR1, CCN1, CCN2, DHFR, G6PD, GRB2, NQO2, PRDX1, PRDX6, STAT3
Go:0042743	Hydrogen peroxide metabolic process	CAT, PRDX1, PRDX6, STAT3
Go:0045454	Cell redox homeostasis	GCLC, PRDX1, PRDX6
Go:0042744	Hydrogen peroxide catabolic process	CAT, PRDX1, PRDX6
Go:0098869	Cellular oxidant detoxification	CAT, DHFR, MAPRE2, PRDX1, PRDX6
Go:2000377	Regulation of reactive oxygen species metabolic process	ARG2, CBR1, DHFR, G6PD, GRB2, NQO2, STAT3
Go:0004601	Peroxidase activity	CAT, MAPRE2, PRDX1, PRDX6
Go:0009409	Response to cold	ATP2B1, HSP90AA1, LCP1, VGF
Go:0019915	Lipid storage	EHD1, ENPP1, LCP1, OSBPL8
Go:0010883	Regulation of lipid storage	EHD1, LCP1, OSBPL8
Go:0030730	Sequestering of triglyceride	ENPP1, LCP1, OSBPL8

ID	Term	Associated Genes Found
Go:1900076	Regulation of cellular response to insulin stimulus	ATP2B1, ENPP1, LCP1, OSBPL8, SORBS1
Go:0032869	Cellular response to insulin stimulus	ATP2B1, BAIAP2L1, ENPP1, GCLC, GOT1, GRB2, LCP1, OSBPL8, SORBS1, SYAP1
Go:0046626	Regulation of insulin receptor signaling pathway	BAIAP2L1, ENPP1, OSBPL8, SORBS1
Go:0046324	Regulation of glucose import	ENPP1, OSBPL8, SORBS1
Go:0031109	Microtubule polymerization or depolymerization	ARL3, CKAP2, HSPA1A, MAPRE2, METAP1, NDRG1
Go:0070507	Regulation of microtubule cytoskeleton organization	ARL3, CKAP2, HSPA1A, MAPRE2, METAP1, NDRG1, TACC3
Go:0044380	Protein localization to cytoskeleton	CEP131, MAPRE2, METAP1
Go:0090224	Regulation of spindle organization	HSPA1A, NDRG1, TACC3
Go:0031110	Regulation of microtubule polymerization or depolymerization	ARL3, CKAP2, HSPA1A, MAPRE2, METAP1, NDRG1
Go:0031112	Positive regulation of microtubule polymerization or depolymerization	ARL3, HSPA1A, NDRG1
Go:0031113	Regulation of microtubule polymerization	ARL3, HSPA1A, NDRG1
Go:0032273	Positive regulation of protein polymerization	ARFIP1, ARL3, BAIAP2L1, GRB2, HSP90AA1, HSPA1A, NDRG1
Go:0060236	Regulation of mitotic spindle organization	HSPA1A, NDRG1, TACC3
Go:0072698	Protein localization to microtubule cytoskeleton	CEP131, MAPRE2, METAP1
Go:0090307	Mitotic spindle assembly	HSPA1A, NDRG1, RACGAP1
Go:0046530	Photoreceptor cell differentiation	ARL3, MAPRE2, STAT3
Go:0031116	Positive regulation of microtubule polymerization	ARL3, HSPA1A, NDRG1
Go:2001057	Reactive nitrogen species metabolic process	ARG2, DDAH1, DDAH2, GLA, HSP90AA1
Go:0046209	Nitric oxide metabolic process	ARG2, DDAH1, DDAH2, GLA, HSP90AA1
Go:0051341	Regulation of oxidoreductase activity	DDAH1, DDAH2, DHFR, GLA, HSP90AA1, NOSIP, PDP1
Go:0080164	Regulation of nitric oxide metabolic process	DDAH1, DDAH2, GLA, HSP90AA1
Go:0006809	Nitric oxide biosynthetic process	ARG2, DDAH1, DDAH2, GLA, HSP90AA1
Go:0032768	Regulation of monooxygenase activity	DDAH1, DDAH2, DHFR, GLA, HSP90AA1, NOSIP
Go:1904407	Positive regulation of nitric oxide metabolic process	DDAH1, DDAH2, HSP90AA1
Go:0008652	Cellular amino acid biosynthetic process	DHFR, GLS, GOT1
Go:0009063	Cellular amino acid catabolic process	ARG2, DDAH1, DDAH2, GCDH, GLS, GOT1, HIBCH
Go:0045428	Regulation of nitric oxide biosynthetic process	DDAH1, DDAH2, GLA, HSP90AA1

ID	Term	Associated Genes Found
Go:0043648	Dicarboxylic acid metabolic process	DHFR, GCLC, GLS, GOT1, L2HGDH, SDHA, SDHAF3
Go:0045429	Positive regulation of nitric oxide biosynthetic process	DDAH1, DDAH2, HSP90AA1
Go:0050999	Regulation of nitric-oxide synthase activity	DDAH1, DDAH2, DHFR, GLA, HSP90AA1, NOSIP
Go:0009064	Glutamine family amino acid metabolic process	ARG2, DDAH1, DDAH2, GCLC, GLS, GOT1
Go:1901606	Alpha-amino acid catabolic process	ACADSB, ARG2, DDAH1, DDAH2, GCDH, GLS, GOT1
Go:0006527	Arginine catabolic process	ARG2, DDAH1, DDAH2
Go:0006525	Arginine metabolic process	ARG2, DDAH1, DDAH2
Go:0006536	Glutamate metabolic process	GCLC, GLS, GOT1
Go:0009065	Glutamine family amino acid catabolic process	ARG2, DDAH1, DDAH2, GLS, GOT1

Table S3: Minimum Information about a Flow Cytometry Experiment (MIFlowCyt).

1. **Experiment Overview**	**Details**
1.1. Purpose	To analyze cell cycle and cell death mechanisms of ACY-1215 treated OMM2.5 cells using flow cytometry.
1.2. Keywords	Cell cycle, Apoptosis, Yo-Pro™1, Propidium Iodide
1.3. Experiment Variables	OMM2.5 cells were treated with either 0.5% DMSO, 50 µM Etoposide, 10, 20 and 50 µM ACY-1215 or 20 µM Dacarbazine. Drug treated samples were analyzed at 4, 24 and 96 hours. Experiments were performed in triplicate and quadruplicate.
1.4. Organization Name and Address	Conway Institute, University College Dublin, Belfield, Dublin 4, Ireland
1.5. Primary Contact Name and Email Address	Breandán Kennedy, brendan.kennedy@ucd.ie<br>Husvinee Sundaramurthi, Husvinee.sundaramurthi@ucd.ie
1.6. Date	October 2020 - December 2020
1.7. Conclusions	ACY-1215 treatment arrested cell cycle in the S phase by 24 hours post treatment. A time and dose-dependent increase in apoptotic and late-stage apoptotic cells with a decrease in percentage of viable cells was observed in ACY-1215 treated OMM2.5 cells.
1.8. Quality Control Measures	Etoposide was used as a control for apoptosis analysis, Dacarbazine was used as a clinical control to compare against our drug of interest (ACY-1215) and vehicle control was used in each experiment. Experiments were independently performed 3 to 4 times.<br>BD Accuri™ C6 Flow Cytometer was routinely calibrated according to manufacturer's instructions.
1.9. Other Relevant Experiment Information	Not Applicable.
2. **Flow Sample/Specimen Details**	
**Sample/Specimen Material Description**	**Details**
2.1.1.1. Sample/Specimen Material description	Drug treated live and post-fixed OMM2.5 cells were collected for apoptosis and cell cycle analysis, respectively, as described in Methods section.
2.1.1.2. Biological Sample Source Description<br><br>2.1.1.3. Biological Sample Source Organism Description	Established metastatic UM cell line - OMM2.5 was kindly provided by Dr. Martine Jager, Leiden, The Netherlands (Jager, M.J.; Magner, J.A.; Ksander, B.R.; Dubovy, S.R. Uveal Melanoma Cell Lines: Where do they come from? (An American Ophthalmological Society Thesis). Trans Am Ophthalmol Soc 2016, 114, T5.).

2.1.1.4. Other Relevant Biological Sample Information	
2.1.2. Environmental sample	Not Applicable.
2.1.3. Other Samples	Not Applicable.
2.2 Sample Characteristics	Drug treated OMM2.5 cells were labeled with PI for cell cycle analysis and Yo-Pro1/PI for apoptosis analysis. Cells undergoing different stages of apoptosis, alive and dead cells are distinguished based on intensity of fluorescence.
2.3. Sample Treatment Description	Single cell suspensions were prepared after treatment for 4, 24 or 96 hours with desired drug solutions. Samples were divided into 2 aliquots of 300 µl cell suspension solution. One aliquot of live cells was labeled with Yo-Pro1/PI for apoptosis analysis. While the 2nd tube was fixed with 70% ethanol at 4^{0}C for a few days. Post-fixed cells were then labelled with PI prior to analysis of cell cycle. A maximum of 50,000 events were recorded per sample for analysis.
2.4. Fluorescence Reagent Description	YO-PRO™-1 Iodide : Molecular Probes^{TM} by Life Technologies<br>Propidium iodide (PI) : Molecular Probes^{TM} by Life Technologies
**3. Instrument Details**	
3.1. Instrument Manufacturer	BD Biosciences (https://www.bdbiosciences.com/en-gb)
3.2. Instrument model	BD Accuri^{TM} C6 Flow Cytometer
3.3. Instrument configuration and settings<br><br>3.3.1. Flow Cell and Fluidics<br><br>3.3.2. Light Sources<br><br>3.3.3. Excitation Optics Configuration<br><br>3.3.4. Optical Filters<br><br>3.3.5. Optical Detectors<br><br>3.3.6. Optical Paths	
3.4. Other Relevant Instrument Details	
**4. Data Analysis Details**	
4.1. List-mode Data Files	FCS data files can be obtained upon request to corresponding author, post publication.

4.2. Compensation Description	No compensation was applied
4.3. Data Transformation Details	No transformation was applied
4.4. Gating Details<br><br>4.4.1. Gate description<br>4.4.2. Gate statistics<br>4.4.3. Gate boundaries<br>4.4.4. Other Relevant Gate Information	A representative figure and statistical analysis have been provided in Figures X, X and Supplementary Figures X, X. For additional info please refer to Results and Methods text in manuscript.
